# The heme scavenger hemopexin protects against lung injury during aspergillosis by mitigating release of neutrophil extracellular traps

**DOI:** 10.1172/jci.insight.189151

**Published:** 2025-04-15

**Authors:** Ganlin Qu, Henrique A.L. Ribeiro, Angelica L. Solomon, Luis Sordo Vieira, Yana Goddard, Nickolas G. Diodati, Arantxa V. Lazarte, Matthew Wheeler, Reinhard Laubenbacher, Borna Mehrad

**Affiliations:** Division of Pulmonary, Critical Care, and Sleep Medicine, University of Florida, Gainesville, Florida, USA.

**Keywords:** Immunology, Infectious disease, Pulmonology, Fungal infections, Innate immunity, Neutrophils

## Abstract

Invasive aspergillosis is characterized by lung hemorrhage and release of extracellular heme, which promotes fungal growth. Heme can also mediate tissue injury directly, and both fungal growth and lung injury may induce hemorrhage. To assimilate these interdependent processes, we hypothesized that, during aspergillosis, heme mediates direct lung injury independent of fungal growth, leading to worse infection outcomes, and the scavenger protein hemopexin mitigates these effects. Mice with neutropenic aspergillosis developed a time-dependent increase in lung extracellular heme and a corresponding hemopexin induction. Hemopexin deficiency resulted in markedly increased lung injury, fungal growth, and lung hemorrhage. Using a computational model of the interactions of *Aspergillus*, heme, and the host, we predicted a critical role for heme-mediated generation of neutrophil extracellular traps (NETs) in this infection. We tested this prediction using a fungal strain unable to grow at body temperature and found that extracellular heme and fungal exposure synergized to induce lung injury by promoting NET release, and disruption of NET was sufficient to attenuate lung injury and fungal burden. These data implicate heme-mediated NETosis in both lung injury and fungal growth during aspergillosis, resulting in a detrimental positive feedback cycle that can be interrupted by scavenging heme or disrupting NETs.

## Abstract 2

Invasive pulmonary aspergillosis results hemorrhage and heme release, promoting fungal growth and lung injury via NETosis. Hemopexin, and the distruption of NETs, mitigate these effects.

## Graphical abstract

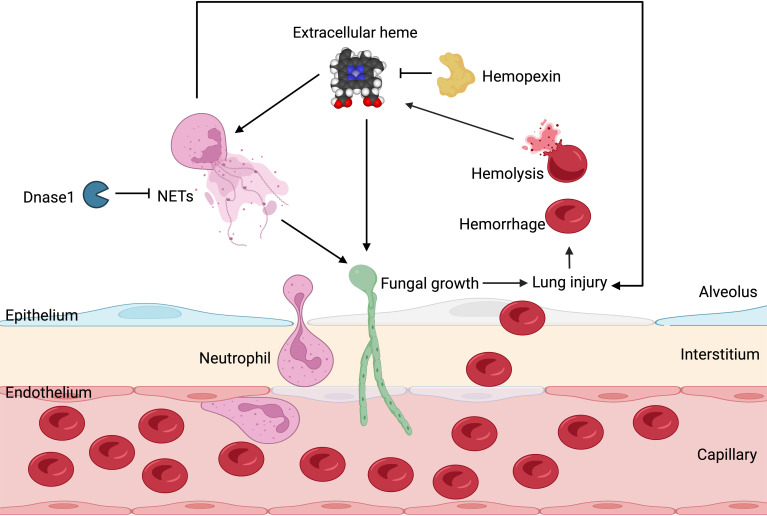

## Introduction

Invasive pulmonary aspergillosis is a common fungal pneumonia of immunocompromised hosts and carries high mortality (1). The spores of the fungus are ubiquitous in air and are inhaled daily by all humans. Normal hosts clear these inhaled spores without becoming ill, but in individuals with impaired immunity, the spores germinate into hyphal filaments that penetrate the lung epithelium, cause pneumonia, and can then disseminate to other organs. Aspergillosis is becoming more common and more resistant to antifungal drugs, prompting WHO to categorize it as a critical priority for research and public health (2).

Pulmonary aspergillosis has long been recognized to result in lung hemorrhage, both in the human infection and in rodent models, thought to result from the physical disruption of blood vessel walls by the invading fungal hyphae (3–5). This hemorrhage causes local hemolysis: the lysis of extravascular red blood cells releases hemoglobin into the extracellular space, which is then degraded to release extracellular heme (6, 7). We recently reported that extracellular heme is utilized by *Aspergillus* as a source of iron, rendering the pathogen more virulent and worsening the outcome of the infection (5). In addition, however, extracellular heme is an endogenous danger signal — an alarmin — that interacts with the host cells via multiple context-dependent mechanisms. Conversely, the host mitigates these injurious effects by scavenging extracellular heme with the liver-derived plasma acute-phase protein, hemopexin (6). The role of extracellular heme and its clearance in progression of aspergillosis and associated tissue injury is only beginning to be understood.

A notable challenge to defining this biology is its complexity: The outcome of aspergillosis is determined by the interplay of multiple processes that unfold over time, including fungal growth, fungal killing by the host, hemorrhage and local hemolysis, heme sequestration, the effects of heme on both the fungus and host cells, and the development of lung injury, which can, itself, cause more hemorrhage. These mechanisms relate to each other by feedback, feed-forward, and redundant loops that can progress in parallel or in series, thus producing a complex network. Mechanistic computational modeling is a powerful tool that is well suited to the systematic integration of such complex biological mechanisms and is more efficient than serial testing of hypotheses focused on individual nodes in the network. A computational model, based on experimental data, provides a dynamic simulation of the system that can be used to generate novel hypotheses and serve as an in silico laboratory to assess predicted outcomes of hypothetical scenarios. We previously generated a multiscale agent-based model of aspergillosis, explicitly representing the infected lung in 3 dimensions, within which cells reside, move, and interact with different molecular species (8–10). In the current manuscript, we used an expansion of this computational model, in concert with experimental approaches, to test the hypothesis that, during aspergillosis, heme mediates direct lung injury independent of fungal growth, leading to worse infection outcomes, and the scavenger protein hemopexin mitigates these effects.

## Results

### Progressive increase in heme and hemopexin during invasive pulmonary aspergillosis.

**Figure 1:**
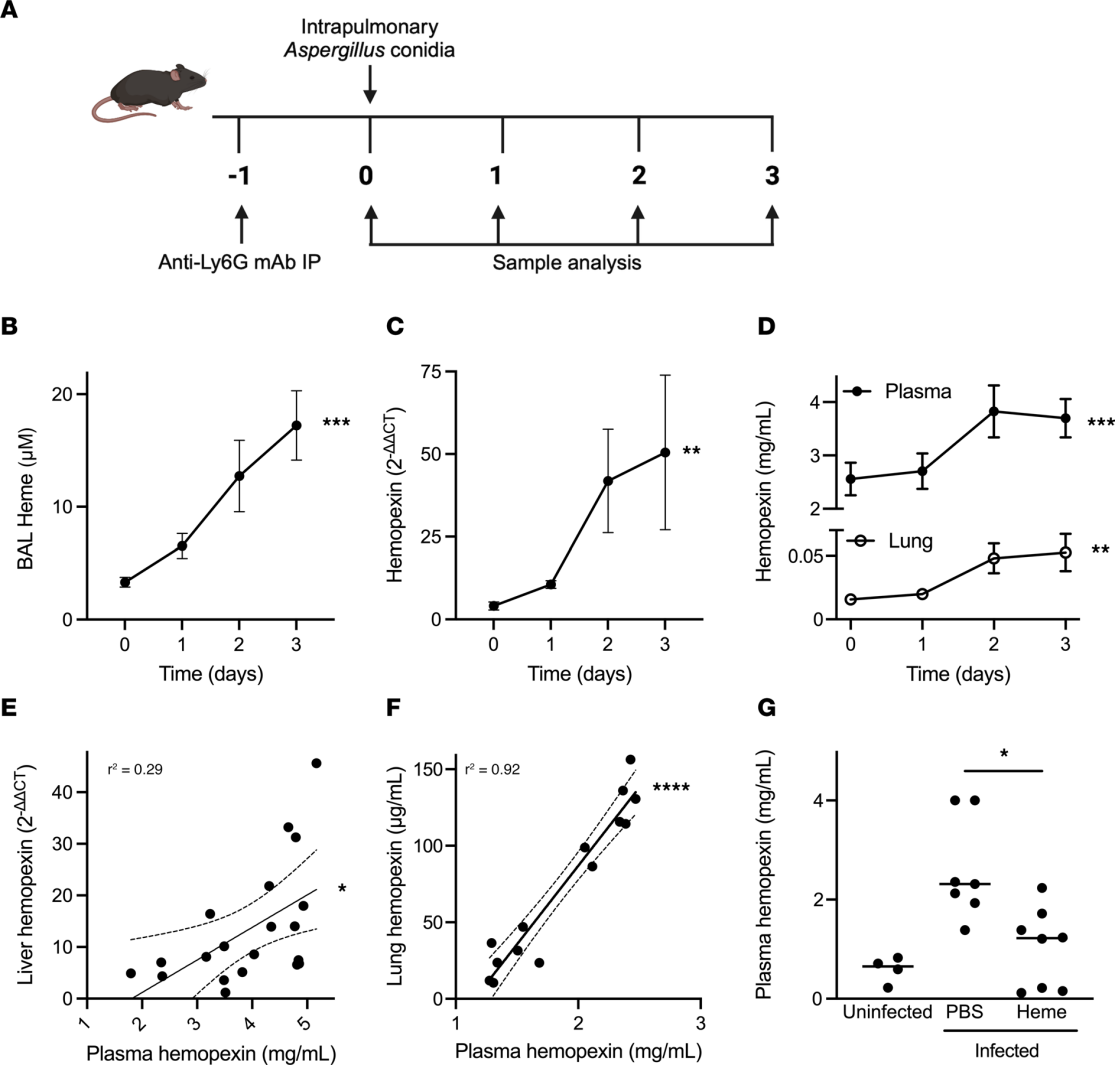
Lung heme content and hemopexin during invasive pulmonary aspergillosis. (**A**) Schematic representation of the experimental protocol. Image made in BioRender. (**B**) The concentration of heme in bronchoalveolar lavage (BAL) over the first 3 days of invasive aspergillosis. (**C** and **D**) Time series of liver hemopexin transcription and plasma and lung hemopexin protein concentrations. In panels **B**–**D**, values represent mean and standard error of the mean (SEM) of *n* = 8–11 animals per time point or per group in each panel, and time 0 indicates neutropenic but uninfected animals. (**E** and **F**) Correlation of liver hemopexin transcription and plasma hemopexin protein and of lung and plasma hemopexin concentration. The solid line represents the linear regression, and dashed lines represent the 95% confidence intervals. These panels show a reanalysis of the data in panels **C** and **D**. (**G**) Effect of exogenous administration of heme on plasma hemopexin during aspergillosis. Intrapulmonary administration of heme was performed on day 1 of infection, and plasma hemopexin was measured on day 2 of infection. Dots represent individual animals and horizontal lines represent medians. Each figure represents pooled data from 2 independent experiments. *, **, ***, and **** denote *P* values of <0.05, <0.01, <0.001, and <0.0001, respectively. Statistical tests: **B**–**D**, 1-way ANOVA; **E** and **F**, simple linear regression; **G**, Kruskal-Wallis 1-way ANOVA with Dunn’s multiple-comparison test.

We began by measuring the lung extracellular heme content during invasive aspergillosis in neutropenic wild-type mice (Figure 1A) and found a time-dependent increase in bronchoalveolar lavage (BAL) extracellular heme with aspergillosis (Figure 1B), which paralleled the extent of lung hemorrhage, which we reported in a prior publication (5). The transcription of hemopexin, a liver-derived acute-phase protein that scavenges extracellular heme, was similarly upregulated (Figure 1C). Both the plasma and lung hemopexin concentration increased over time, with lung concentration being an order of magnitude lower than plasma (Figure 1D). Liver hemopexin transcription correlated with plasma hemopexin concentration (Figure 1E), and plasma and lung concentrations correlated (Figure 1F) — consistent with synthesis of hemopexin in the liver, circulation in plasma, and diffusion of plasma hemopexin into the lungs. To test whether plasma hemopexin scavenges extracellular heme in infected lungs, we measured plasma hemopexin concentration after intrapulmonary administration of exogenous heme to animals with experimental aspergillosis. The amount of heme present in approximately 15 μL of whole blood was provided to each mouse. Intrapulmonary administration of heme on day 1 of infection resulted in lower plasma hemopexin on day 2 (Figure 1G). Taken together, these data suggest that, during invasive aspergillosis, hemopexin synthesis is upregulated in the liver, then reaches the lungs via the circulation, where it scavenges extracellular heme.

### Hemopexin is essential to host defense in invasive aspergillosis.

**Figure 2:**
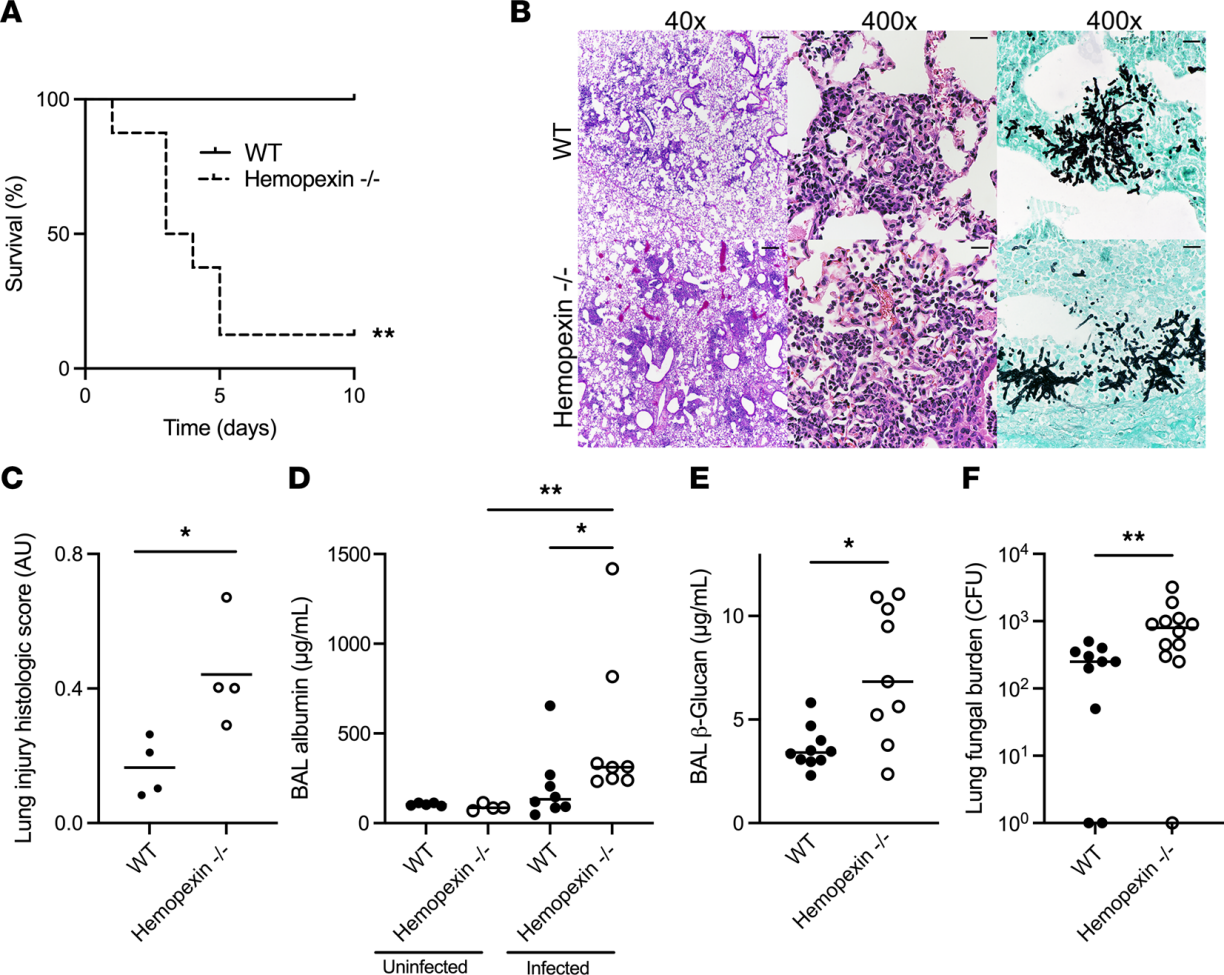
Protective role of hemopexin in invasive pulmonary aspergillosis. (**A**) Survival of neutropenic wild-type and hemopexin-deficient mice with invasive aspergillosis. *n* = 6–8 per group. (**B**) Lung histology of wild-type and hemopexin-deficient mice on day 3 of infection. Hematoxylin and eosin and Gomori methenamine silver stains. Original magnifications are indicated. Scale bars are 200 μm and 20 μm long in the 40× and 400× micrographs, respectively. (**C**) Histologic score of lung injury in neutropenic mice with aspergillosis. (**D**) Extent of lung injury, measured as BAL fluid albumin concentration, in neutropenic mice with or without aspergillosis. (**E** and **F**) Lung fungal content, measured as BAL fluid (1→3)-β-d-glucan content and lung colony-forming units (CFU), in neutropenic mice with aspergillosis. In panels **C**–**F**, dots represent individual animals and horizontal lines represent medians. Since 0 cannot be depicted on a log scale, animals with no fungal growth are depicted as having 1 CFU in panel **F**. AU, arbitrary units. Each figure represents pooled data from 2 independent experiments, except the histology, which represents data from 3 independent experiments. * and ** denote *P* values of <0.05 and <0.01, respectively. Statistical tests: **A**, log-rank test; **C**, **E**, and **F**, 2-tailed Mann-Whitney; **D**, Kruskal-Wallis 1-way ANOVA with Dunn’s multiple-comparison test.

To determine the contribution of hemopexin to host defense in aspergillosis, we next infected neutropenic wild-type and hemopexin-deficient mice with *Aspergillus* conidia. Hemopexin-deficient mice had dramatically increased mortality (Figure 2A). To mitigate survivorship bias in hemopexin-deficient mice, we reduced the infectious inoculum in subsequent experiments. The lungs of neutropenic wild-type and hemopexin-deficient mice were normal in the absence of infection (Supplemental Figure 1; supplemental material available online with this article; https://doi.org/10.1172/jci.insight.189151DS1). On day 3 of infection, both wild-type and hemopexin-deficient mice displayed growth of fungal hyphae in the lung, associated with areas of leukocyte infiltration and lung injury (Figure 2B). The extent of lung injury, as seen on histology, was more severe in hemopexin-deficient than wild-type mice in the context of infection (Figure 2C). To further quantify the extent of lung injury, we measured BAL albumin and found that hemopexin-deficient animals had normal lung barrier integrity at baseline but increased permeability during the infection (Figure 2D). This increased degree of lung injury in hemopexin-deficient mice was associated with higher lung fungal burden, as measured by the lung content of (1→3)-β-d-glucan, a major fungal cell wall carbohydrate released from the growing hyphae (11), and lung colony-forming units (CFU) (Figure 2, D and E) yet similar number of lung leukocytes (Supplemental Figure 2). Furthermore, administration of hemopexin to infected hemopexin-deficient mice resulted in lower lung fungal burden (Supplemental Figure 3). Taken together, these data indicate that neutropenic hemopexin-deficient mice develop more severe aspergillosis than wild-type counterparts.

**Figure 3:**
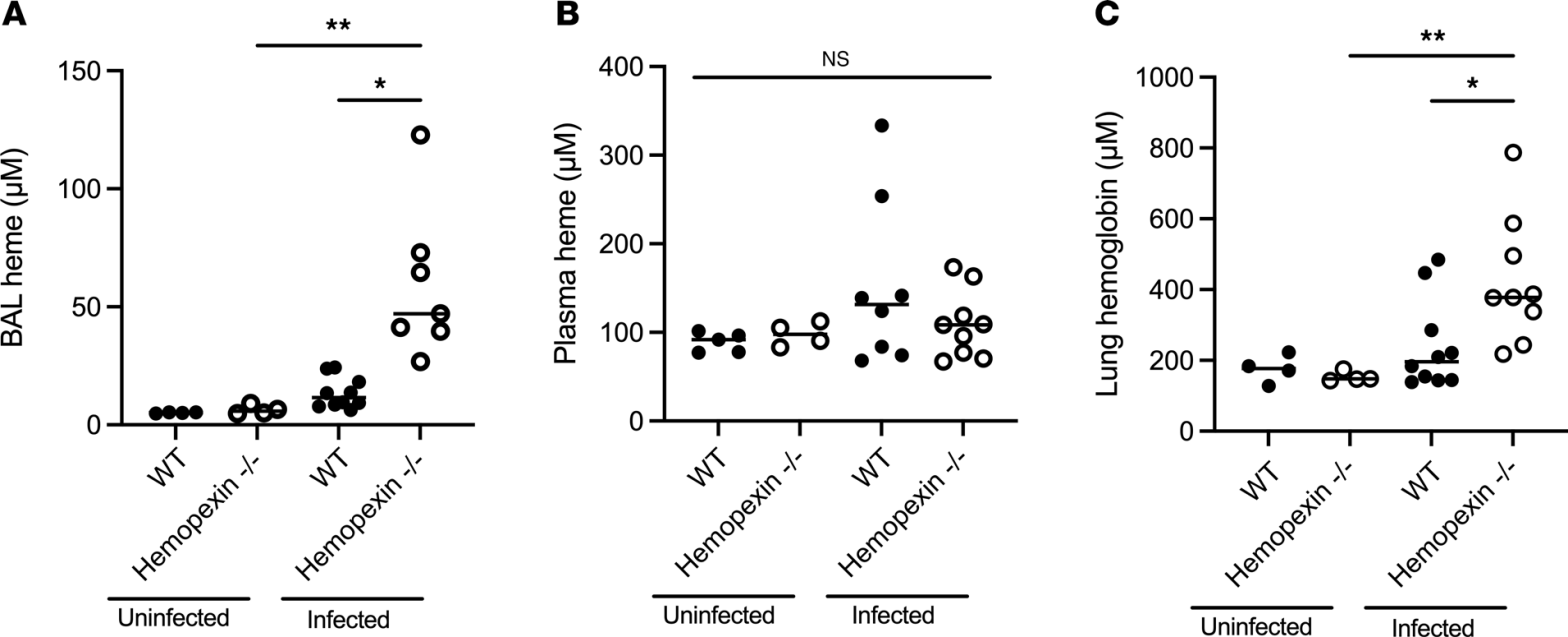
The effect of hemopexin on lung heme and hemoglobin accumulation in aspergillosis. (**A** and **B**) BAL and plasma heme concentrations in wild-type and hemopexin-deficient neutropenic mice with or without aspergillosis. (**C**) Lung hemoglobin concentration in wild-type and hemopexin-deficient neutropenic mice with or without aspergillosis. Dots represent individual animals and horizontal lines represent medians. Each figure represents pooled data from 2 independent experiments. * and ** denote *P* values of <0.05 and <0.01, respectively. Statistical tests: **A**–**C**, Kruskal-Wallis 1-way ANOVA with Dunn’s multiple-comparison test.

We next assessed the effect of hemopexin on local lung hemolysis during aspergillosis. As expected, there was no difference between uninfected wild-type and hemopexin-deficient mice in lung heme and hemoglobin content, and lung heme content was higher in infected hemopexin-deficient mice as compared with wild-type (Figure 3A). This hemolysis was localized to the lung, since the concentration of blood free heme did not differ between groups (Figure 3B). Surprisingly, the lung concentration of extracellular hemoglobin was also elevated in the lungs of infected hemopexin-deficient animals (Figure 3C), suggesting that the presence of extracellular heme in the infected lung promotes further hemorrhage.

### Computational modeling of the role of heme in pulmonary aspergillosis.

**Figure 4:**
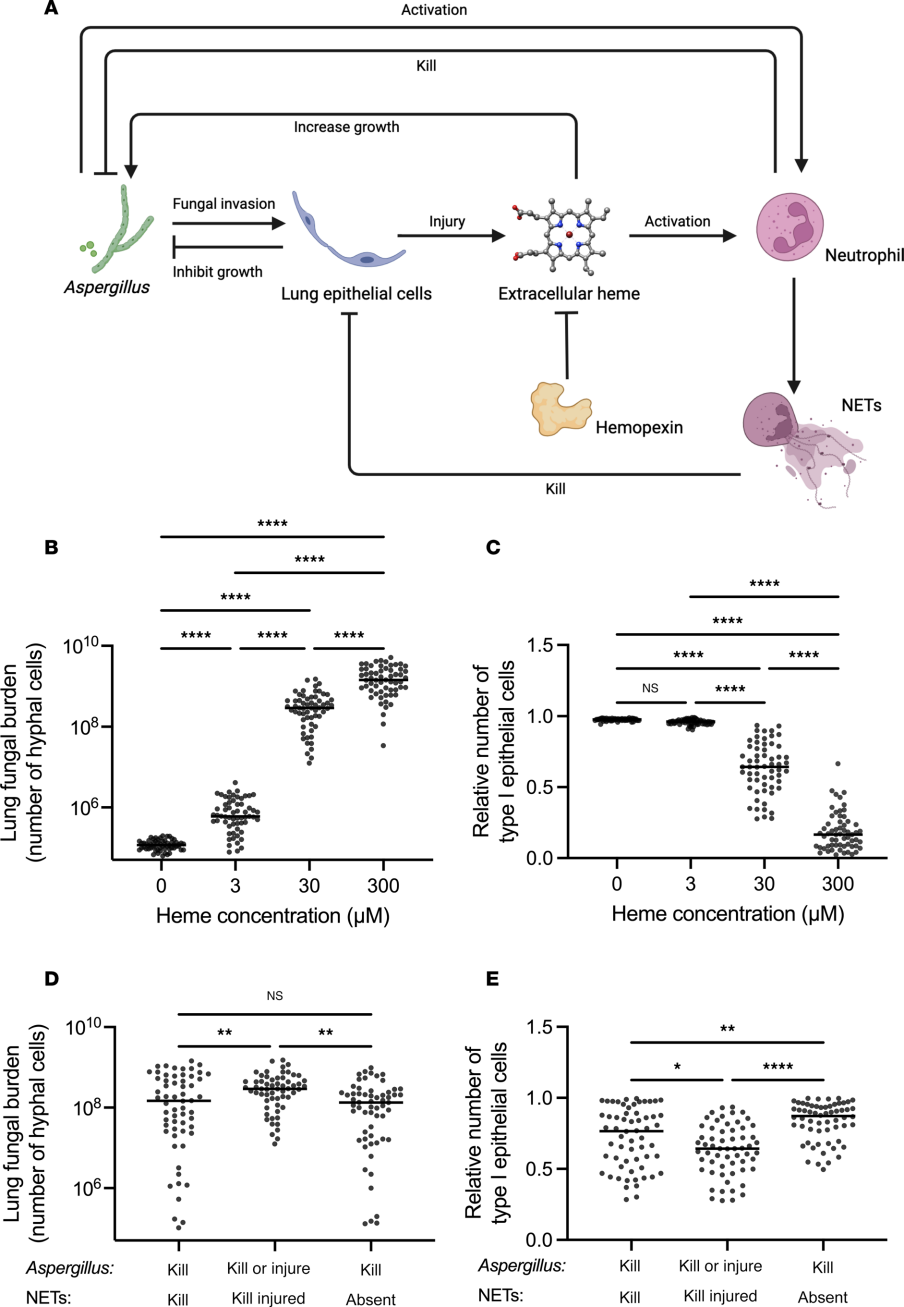
Computational model of the role of heme in aspergillosis. (**A**) Schematic representation of key mechanisms incorporated into the mathematical model. Image made in BioRender. NET, neutrophil extracellular trap. (**B** and **C**) The predicted effect of different concentrations of alveolar extracellular heme on lung fungal load and lung injury. Lung CFU was represented as the number of hyphal fragments between septa, and lung injury is represented as death of type I alveolar epithelial cells compared with baseline representation of the lung. (**D** and **E**) Comparison of the predicted effects of NETs on lung fungal burden and injury, in the presence of 30 μM heme. *X* axis labels describe the model rules regarding effects of *Aspergillus* and NETs on type I alveolar epithelial cells. Dots represent results of simulation runs and horizontal lines represent medians. *, **, and **** denote *P* values of <0.05, <0.01, and <0.0001, respectively. Statistical tests: **B**–**E**, Kruskal-Wallis 1-way ANOVA with Dunn’s multiple-comparison test.

We reasoned that several mechanisms can result in the increased lung hemorrhage in hemopexin-deficient mice during aspergillosis: These mechanisms include the effects of extracellular heme on the fungus, promoting its growth and pathogenicity; the effects of extracellular heme on the host, promoting lung injury; or a combination of these. To capture these possibilities, we incorporated several new mechanisms, including hemorrhage and heme release, into a previously generated computational model of the immune response against *A*. *fumigatus* (9). The resulting model (a simplification of which is presented in Figure 4A) was constructed and parameterized using the published literature and data from the current manuscript (see Supplemental Data 1 for details). The model simulates mice with partial neutrophil depletion, by reducing the number of lung neutrophils to the experimentally observed number in animals with antibody-mediated partial neutrophil depletion (Supplemental Figure 4). The model does not explicitly include hemopexin; instead, we vary the heme quantity in the alveoli to reproduce the amounts of heme observed in wild-type and hemopexin-deficient mice during the infection. We incorporated NETs into the computational model, since neutrophils are pivotal in host defenses against *Aspergillus*, both heme and *Aspergillus* induce NET formation (12, 13), and NETs can mediate lung injury (14–16). We simulated the effect of DNase in the model by reducing the amount of intra-alveolar NETs. Using the computational model, we assessed the effects of lung extracellular heme on the outcome of the infection by running 48 hours of simulated infection in partially neutropenic mice with differing concentrations of extracellular heme in the alveoli. We chose the range of simulated alveolar heme concentrations based on Figure 1B and Figure 3A, accounting for dilution of alveolar contents during bronchoalveolar lavage. The model predicted a positive correlation between alveolar heme concentrations and lung fungal burden (Figure 4B). The model further predicted that increasing concentrations of intra-alveolar heme resulted in progressively greater degree of lung injury, represented in the model by the loss of type I alveolar epithelial cells (Figure 4C).

We next assessed the predicted effect of NETs during the infection, by comparing the predicted effect of 3 hypothetical rules on the loss of type I alveolar epithelial cells (and hence the development of lung injury): In the first condition, NETs and hyphae could each kill epithelial cells independently. In the second condition, hyphae could kill epithelial cells independently, and NETs could only synergistically kill epithelial cells that had previously been injured by hyphae. In the third condition, no NETs were present, and epithelial cell killing was only mediated by hyphae. As expected, the model predicted that synergistic killing results in the most, and absence of NETs results in the least, alveolar epithelial cell death. Interestingly, the model also predicted that synergistic killing results in higher lung fungal burden that was mitigated if NETs were not present (Figure 4, D and E).

### Hemopexin protects against aspergillosis-induced lung injury independent of fungal growth.

**Figure 5:**
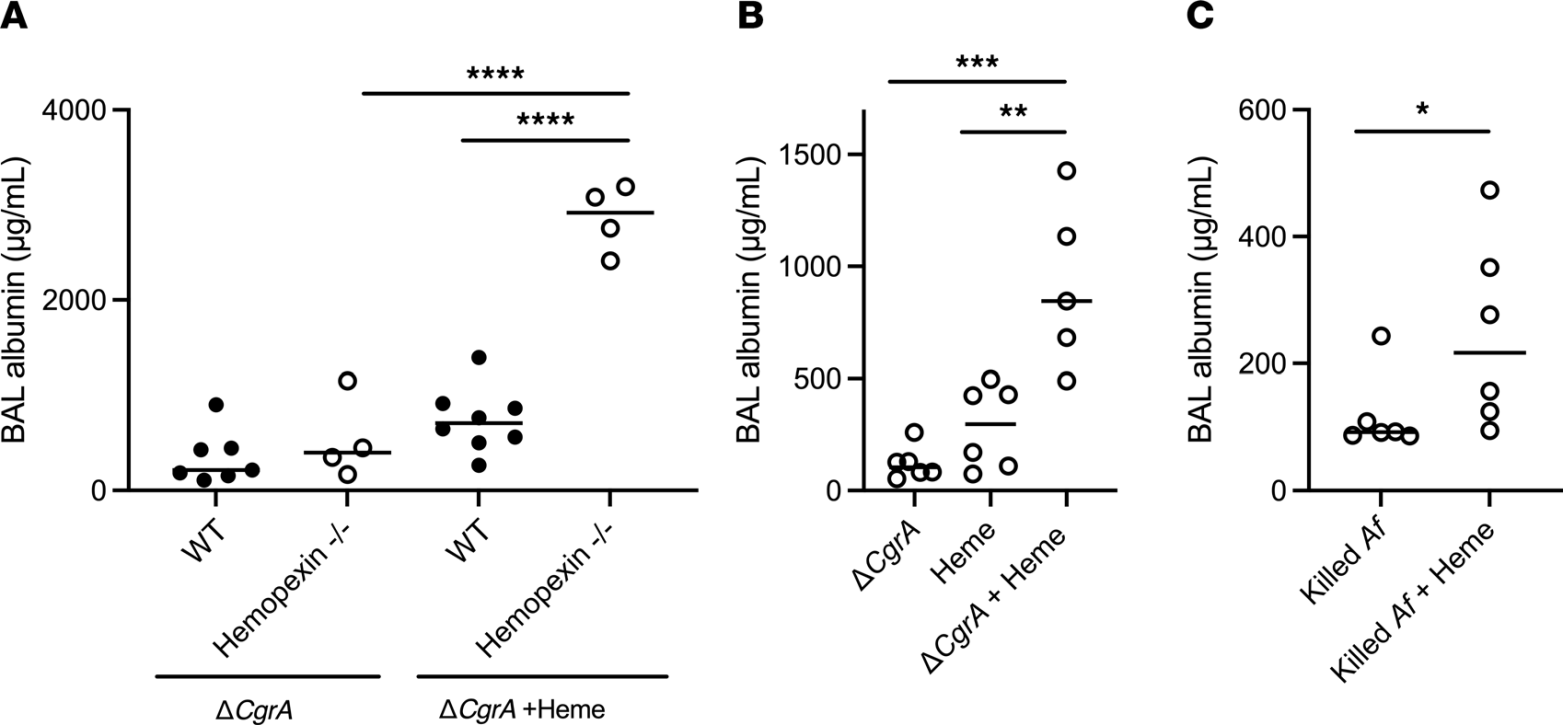
The role of heme in the induction of lung injury in neutropenic aspergillosis. (**A**) Lung injury in neutropenic wild-type and hemopexin-deficient mice challenged with germlings of Δ*CgrA* (temperature intolerant) strain, with or without intrapulmonary administration of heme. (**B**) Effect of exogenous heme on lung injury in neutropenic hemopexin-deficient mice challenged with Δ*CgrA* germlings, heme, or both. (**C**) Effect of exogenous heme on lung injury in neutropenic hemopexin-deficient mice challenged with ethanol-killed *Aspergillus* germlings, with or without heme. Dots represent individual animals and horizontal lines represent medians. Each figure represents pooled data from 2 independent experiments. *, **, ***, and **** denote *P* values of <0.05, <0.01, <0.001, and <0.0001, respectively. Statistical tests: **A** and **B**, Kruskal-Wallis 1-way ANOVA with Dunn’s multiple-comparison test; **C**, 2-tailed Mann-Whitney.

The mathematical model predicted that extravascular heme in the alveolus mediates lung injury both by promoting fungal growth and, independently, via a direct effect on the host. To separate the effect of extracellular heme on the fungus from its predicted effects on the host, we next used the Δ*CgrA* temperature-intolerant mutant strain of *Aspergillus*, which grows at room temperature but not at 37°C, and is hence avirulent in vivo (17). We cultured the Δ*CgrA* to the germling stage at room temperature, then used these in intrapulmonary challenges in neutropenic mice. As expected, neither wild-type nor hemopexin-deficient neutropenic mice developed appreciable lung injury after challenge with Δ*CgrA* germlings (Figure 5A). In contrast, when Δ*CgrA* germlings were coadministered with intrapulmonary heme, hemopexin-deficient animals developed lung injury in response to the challenge (Figure 5A). To assess whether the latter effect is mediated merely by the intrapulmonary administration of exogenous heme to hemopexin-deficient animals, we challenged hemopexin-deficient animals with Δ*CgrA* germlings, heme alone, or both, and found that the combination of heme and Δ*CgrA* germlings was necessary to the development of lung injury in these hosts (Figure 5B). Finally, we found similar results when we challenged hemopexin-deficient mice with killed wild-type *Aspergillus* germlings (Figure 5C). Taken together, these data indicate that, independent of fungal growth, intra-alveolar extracellular heme during aspergillosis mediates lung injury, and hemopexin provides protection against this injury — consistent with the prediction of the computational model.

### Heme mediates poor outcomes in aspergillosis by inducing NETosis.

**Figure 6:**
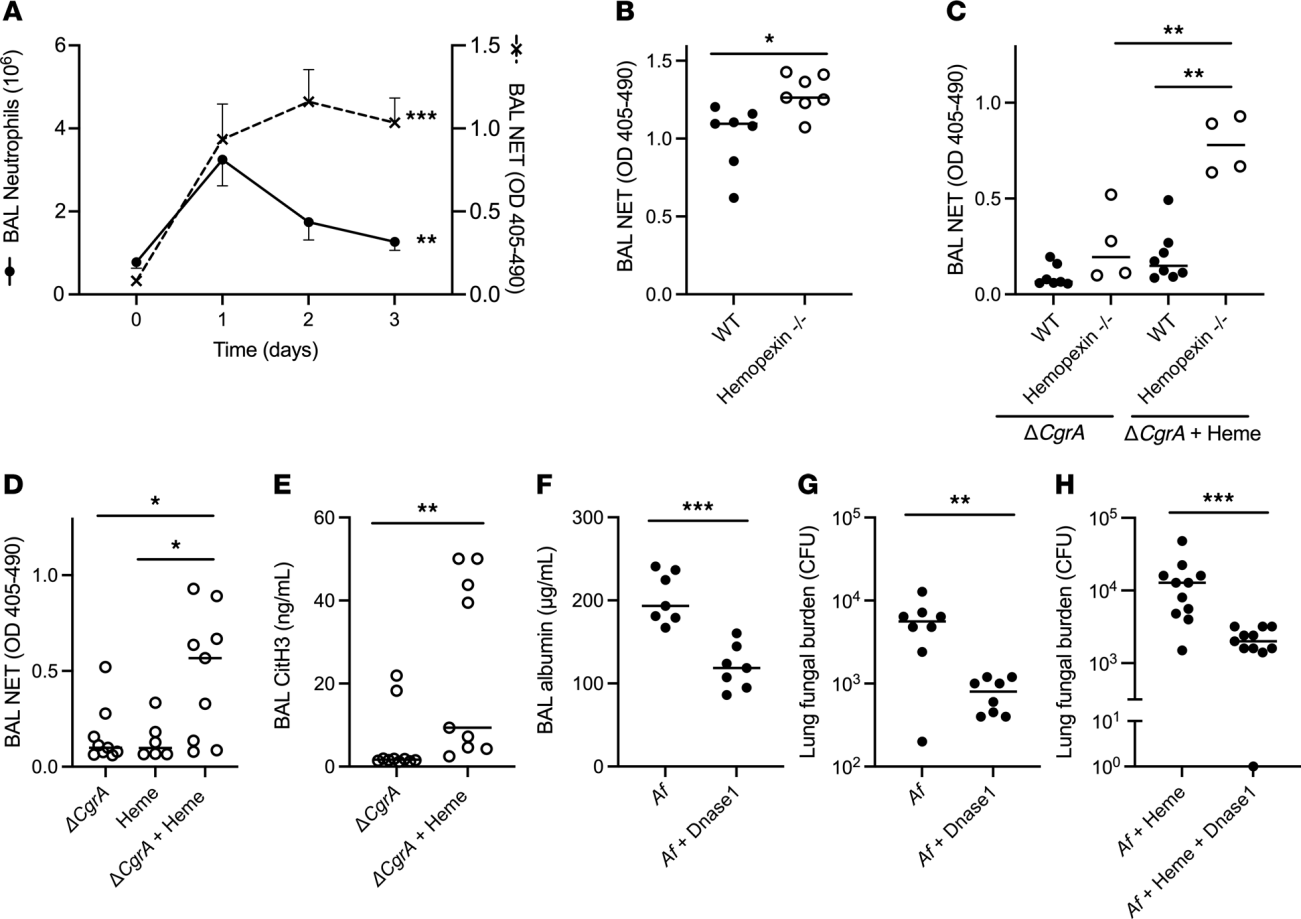
NET formation and resulting lung injury induced by heme, which is attenuated by hemopexin. (**A**) Number of neutrophils and NET level in the lungs of wild-type neutropenic mice during invasive pulmonary aspergillosis. (**B**) BAL NET level of neutropenic mice on day 3 after *Aspergillus* infection. (**C**) NET level in the plasma and BAL of neutropenic mice after challenge with Δ*CgrA* germlings with or without exogenous heme. (**D** and **E**) Effect of exogenous heme on lung NET formation in neutropenic hemopexin-deficient mice challenged with Δ*CgrA* germlings. (**F**–**H**) The effect of DNase treatment on lung injury and fungal burden in wild-type mice with invasive aspergillosis. In **F** and **G**, DNase 1 was administered on day 2 and measurements were taken on day 3 of infection. In **H**, DNase 1 was administered after 12 hours and measurements were taken 24 hours after onset of infection. Since 0 cannot be depicted on a log scale, a mouse with no fungal growth is depicted as having 1 CFU in panel **H**. In panel **A**, values represent mean ± SEM of *n* = 5–8 animals per group per time point, and time 0 refers to uninfected neutropenic animals. In panels **B**–**H**, dots represent individual animals and horizontal lines represent medians. Panels **A**–**C**, **F**, and **G** represent pooled data from 2 independent experiments, and panels **D**, **E**, and **H** represent pooled data from 3 independent experiments. *, **, and *** denote *P* values of <0.05, <0.01, and <0.001, respectively. Statistical tests: **A**, 1-way ANOVA; **B** and **D**–**H**, 2-tailed Mann-Whitney; **C**, Kruskal-Wallis 1-way ANOVA with Dunn’s multiple-comparison test.

We next sought to determine the mechanism by which heme mediates lung injury independently of fungal growth. While heme, acting as an alarmin, can directly affect the host in several ways, the mathematical model predicted a detrimental role in heme-mediated NETosis during aspergillosis (Figure 4, D and E). We began to test this prediction by measuring the number of neutrophils and the quantity of NETs in wild-type mice with aspergillosis. We found that, in this partially neutropenic animal model, the number of alveolar neutrophils peaked on day 1 and decreased thereafter; in contrast, the number of alveolar neutrophils was an order of magnitude higher in mice treated with isotype control antibody and challenged with *Aspergillus* (Supplemental Figure 4). In the partially neutropenic mice, we found the quantity of NETs peaked on day 1 but plateaued thereafter (Figure 6A) — consistent with the notion that neutrophils arriving in the lung on days 2–3 of the infection rapidly undergo NETosis.

To determine the contribution of heme and hemopexin to NET production during aspergillosis, we next compared BAL NET quantity in wild-type and hemopexin-deficient mice with invasive aspergillosis and found higher NET levels in hemopexin-deficient mice (Figure 6B). We found similar results in wild-type mice with aspergillosis challenged with exogenous intrapulmonary heme (Supplemental Figure 5A). Furthermore, we found reduced NET formation after intrapulmonary administration of hemopexin to animals with aspergillosis (Supplemental Figure 5B). To determine if this observation is attributable to higher fungal burden in hemopexin-deficient animals (Figure 2, E and F) or the direct effect of heme on the host, we next compared BAL NET levels in wild-type and hemopexin-deficient mice, challenged with Δ*CgrA* germlings, alone or with intrapulmonary heme. Similar to the degree of lung injury under these conditions (Figure 5A), hemopexin-deficient mice generated more intra-alveolar NETs (Figure 6C), an effect that required challenge with both Δ*CgrA* germlings and heme (Figure 6D).

A key feature of NET formation is the citrullination of histones contained in the expelled chromatin, a process mediated by peptidylarginine deiminase enzymes (18). To assess this feature of NETosis in aspergillosis, we compared the concentration of citrullinated histone-3 in the alveoli of hemopexin-deficient mice challenged with Δ*CgrA* germlings, with or without heme. In agreement with the NET assay, we found higher concentrations of citrullinated histone-3 in the lungs of animals challenged with both Δ*CgrA* germlings and heme (Figure 6E). Peptidylarginine deiminase-4 (PAD4, 1 of 5 peptidylarginine deiminase enzymes) is highly expressed in neutrophils, localizes to the nucleus, and has been shown to be necessary to NET formation in some disease contexts (19, 20) but not others (21, 22). To determine whether this mechanism is operational in heme-mediated NETosis in aspergillosis, we compared the extent of lung injury between wild-type and PAD4-deficient mice in the context of neutropenic invasive aspergillosis. We found no difference in BAL albumin concentration or quantity of NETs between these groups (Supplemental Figure 5, C and D), indicating that PAD4 is dispensable to NETosis in the context of aspergillosis.

The mathematical model predicted that the absence of NETs during aspergillosis will result in less lung injury and, unexpectedly, lower fungal burden (Figure 4, D and E). To test these predictions, we examined the effect of intrapulmonary administration of DNase 1 to wild-type mice during invasive aspergillosis. As expected, this treatment resulted in lower concentration of NETs in the BAL (Supplemental Figure 6, A and B). Consistent with the predictions of the mathematical model, DNase treatment also resulted in attenuated lung injury and lower lung fungal burden in wild-type mice (Figure 6, F and G). Since our earlier data showed that lung extracellular heme was critical to NETosis during aspergillosis (Figure 6, C–E), we finally tested the effect of DNase 1 treatment in invasive aspergillosis in the context of exogenous administration of heme and again found a lower lung fungal burden in treated animals (Figure 6H). DNase therapy did not, however, affect lung heme content, consistent with the notion that NETosis is downstream of lung hemorrhage (Supplemental Figure 6, C and D). Collectively, these data implicate heme-mediated NETosis in both lung injury and fungal growth during pulmonary aspergillosis.

## Discussion

The association of aspergillosis with hemorrhage has been documented since the initial descriptions of the infection in the 19th century (23). Histopathologically, *Aspergillus* hyphae invade and disrupt blood vessels, causing intra-alveolar hemorrhage and hemorrhagic and coagulative necrosis in autopsy series (24). We previously reported that *Aspergillus* acquires heme from the abundant extracellular hemoglobin made available by tissue hemorrhage and lysis of red blood cells, rendering it more virulent (5), consistent with the critical dependence of *Aspergillus* species on iron acquisition for their growth (25, 26). In the current work, we found that extracellular heme, independent of its effect on the fungus, also mediates NET formation, which both induces lung injury and impairs fungal killing, and that these effects are mitigated by the heme scavenger hemopexin.

Erythrocytes are the most abundant cell type in the body (27), and their premature destruction — hemolysis — occurs in diverse pathological settings. Following release from erythrocytes, the tetrameric α_2_β_2_ hemoglobin molecules dissociate to αβ heterodimers, which are then bound by the liver-derived acute-phase protein, haptoglobin, and scavenged by mononuclear phagocytes via the receptor, CD163 (28). Once the available haptoglobin is depleted, dimeric extracellular hemoglobin is oxidized to methemoglobin and releases its heme moiety. The resulting extracellular heme is bound by hemopexin, and scavenged via CD91, a receptor expressed by multiple cell types (28). Independent of its cause, hemolysis results in inflammation, mediated by the release of damage-associated molecular patterns, among which labile extracellular heme is the best characterized. In addition, both dimeric hemoglobin and heme induce oxidative stress (29). These mechanisms have been investigated thoroughly in the context of classical hemolytic conditions, such as hemoglobinopathies (30), but may also be relevant to a broad array of other diseases, given the abundance of erythrocytes and prevalence of hemolysis in different pathologic states.

The above mechanisms have been implicated in lung injury caused by systemic hemolysis, for example in sickle cell acute chest syndrome and non-antibody-mediated transfusion-related lung injury (30, 31). Systemic hemolysis occurs in numerous infections by several mechanisms, namely direct pathogen invasion of erythrocytes, elaboration of hemolysins, and immune-mediated processes (32). Many microbes, including *Aspergillus*, can also take up and use heme as a nutrient (33, 34), but the mechanistic contribution of hemolysis to microbial pathogenesis is defined in only a few infections, most notably in malaria and sepsis (35, 36). The current work adds to this literature by defining the role of local (but not systemic) hemolysis in aspergillosis, via a positive feedback loop in which lung hemorrhage and release of extracellular heme both promotes the growth of the pathogen and mediates further lung injury, thus causing further hemorrhage and local hemolysis — and defining the key role of hemopexin in protecting against this deleterious cycle.

Formation of NETs is a canonical effector function that is key to both neutrophil-mediated antimicrobial effects and inflammatory tissue damage (37). Neutrophils release NETs in response to many pathogens, including *Aspergillus* (12, 38). While NET formation is antimicrobial against many pathogens, the role of neutrophil NETs during aspergillosis remains undefined: Specifically, NETs do not improve neutrophil killing of *Aspergillus* (39–41), and reports of the effect of NETosis on inhibiting fungal growth are conflicting (41, 42). On the other hand, NET formation in the lungs can damage the alveolar epithelium and endothelium and propagates lung injury (43, 44). Finally, extracellular heme, acting as an alarmin, is a potent inducer of neutrophil NETs in vitro and in several disease models (13, 45–47). A methodological challenge in experimentally isolating the role of NETosis in vivo is that disruption of mechanisms that lead to NETosis can affect the phenotype of neutrophils in other ways; for example, disruption of NADPH oxidase in neutrophils inhibits NET formation (48) but also results in neutrophil TNF production in response to *Aspergillus* (49). In addition, the mechanisms that initiate NETosis differ between pathogens — for example, we found that PAD4-deficient mice (which have impaired NET production in response to *Staphylococcus aureus* and LPS; refs. 19, 20) had intact NET production during aspergillosis (Supplemental Figure 5, C and D). We dealt with these challenges by enzymatically disrupting NETs after their formation. Our data add to the literature by implicating extracellular heme as a mediator of NETosis during aspergillosis and identifying NETosis as maladaptive and harmful to the host during this infection. In addition to mitigating lung injury, disruption of NETs in our model also resulted in lower lung fungal burden. As suggested by the computational model, we speculate that lung injury may enhance lung hemorrhage and heme release, thus promoting fungal growth; in addition, injury to alveolar epithelial cells and disruption of their antimicrobial functions may further impair fungal clearance.

Computational modeling can help in understanding complex biological systems, by incorporating multiple processes and intertwined feedback loops, and serve as a virtual laboratory to explore possible mechanisms and generate hypotheses to test experimentally. In the current work, we expanded a previously published model that includes many of the components involved in the early immune response to *A*. *fumigatus* (9), incorporating additional mechanisms to examine the role of heme in this infection. From a modeling perspective, this work advances the field in 2 ways. First, it demonstrates how a mathematical model of a disease can be used effectively for the discovery of new biology and the efficient testing of experimental hypotheses in silico before taking them into the laboratory. Second, it provides an example of how the modular design of the model simplifies its expansion, by allowing the addition of new mechanisms (50). We anticipate that, over time, the model will include many aspects of the immune response to a respiratory infection, and for any given question, a sensitivity analysis of model parameters can identify model features that are dispensable in the particular context of the question (51) — thus allowing the model to be adapted to many investigations.

An unexpected feature of aspergillosis-associated lung injury in hemopexin-deficient animals was an apparent dissociation between the extent of lung injury and number of lung leukocytes — specifically, hemopexin-deficient animals developed higher lung fungal burden than wild-type animals and, independently, also a greater extent of lung injury, but similar number of lung leukocytes (Figures 2 and 5 and Supplemental Figure 2). In this context, the number of lung leukocytes represents a sum of their recruitment and local differentiation on the one hand, and their efflux and various forms of cell death, on the other (52, 53). We speculate that the combination of increased lung fungal burden and marked increase in extracellular labile heme in hemopexin-deficient animals may influence several of these mechanisms simultaneously – for example, we found that hemopexin deficiency resulted in a similar number of lung neutrophils during the infection but increased NET formation, suggesting a combination of enhanced recruitment and enhanced NETosis. Notably, heme mediated, and hemopexin protected against, lung injury, even when mice were challenged with the temperature-intolerant *Aspergillus* mutant or dead fungus — indicating that heme-mediated lung injury in this model occurs both by mediating fungal growth and, independently, by inducing inflammatory damage to the alveolar-capillary barrier.

We conclude that labile extracellular heme released from local hemolysis during aspergillosis is at the nexus of a series of pathogenic mechanisms, including enhancing fungal growth and, separately, inducing lung injury via NETosis, with hemopexin acting as a potent defense against these processes. These findings suggest possible avenues for future research. These include mechanistic studies of other erythrocyte-derived alarmins and examining the effects of heme on cells other than neutrophils during aspergillosis. Finally, host-centered interventions, such as administration of heme- or hemoglobin-scavengers and inhibition or disruption of NETs, may augment existing therapies for aspergillosis.

## Methods

### Sex as a biological variable.

Since male and female animals have similar outcomes in invasive aspergillosis, this study was performed in both male and female animals, and aggregate findings are reported.

### Preparation and administration of A. fumigatus.

*A*. *fumigatus* strain 13073 (American Type Culture Collection) was cultured on Sabouraud’s dextrose agar plates at 37°C for 2–3 weeks prior to conidial harvest in 0.1% Tween 80 in phosphate-buffered saline. In some experiments, a temperature-sensitive mutant strain of *A*. *fumigatus* (Δ*CgrA*, a gift from David S. Askew, University of Cincinnati, Cincinnati, Ohio, USA) was cultured at room temperature under phleomycin selection for 6–8 weeks prior to conidial harvest, as described (17). The resulting conidia were filtered through sterile gauze, enumerated using a hemocytometer, and cultured in RPMI 1640 (Corning) containing 1% penicillin-streptomycin (plus 10% FBS for the Δ*CgrA* strain) in a shaking incubator until the development of germlings. In some experiments, the resulting germlings were killed by resuspending them in 70% ethanol, as described (49).

### Animals, in vivo procedures, and sample collections.

Wild-type, hemopexin-deficient, and PAD4-deficient mice, all on C57BL/6J genetic background, were purchased from Jackson Laboratory and propagated and maintained in a specific pathogen–free vivarium. Age- and sex-matched 7- to 12-week-olds were used in experiments. Aspergillosis was induced as previously described (5, 49). Briefly, mice were rendered neutropenic by intraperitoneal injection of 400 μg of anti-Ly6G mAb (clone 1A8, Bio X Cell) 1 day before intratracheal administration of 30 μL sterile PBS containing 7.5 × 10^6^ resting conidia or 6 × 10^5^ germlings, under anesthesia with ketamine and xylazine. In some experiments, hemin (MilliporeSigma) at 2.4 μM in PBS was administered to mice together with *Aspergillus* via the intratracheal route, and 35 μL via the intranasal route on day 1 of infection, under isoflurane anesthesia. In other experiments, 4,000 U of DNase 1 (Roche) or 125 μg of human hemopexin (Athens Research) in 35 μL of PBS was delivered to each mouse intranasally twice a day for 1 day or 2 days of the infection under isoflurane anesthesia. Sample collections were performed as previously described (54, 55).

### Histology.

Lungs were perfused and then fix-inflated with 4% paraformaldehyde in PBS, embedded in paraffin, sectioned, and stained with hematoxylin and eosin and Gomori’s methenamine silver, as previously described (5, 49). For histological scoring, bright-field digital images of the entire section of H&E-stained lung samples were acquired at 400× magnification (Keyence BZ-X810 microscope). Using image analysis software (QuPath open-source software) (56), acute lung injury was scored in 20 randomly generated 100 μm by 100 μm fields that contained at least 50% alveoli per field, by an observer masked to experimental conditions. We used published criteria to score lung injury (57), with a minor modification: Given that the animals were partially neutrophil depleted, we scored the intra-alveolar and interstitial leukocytes, rather than only neutrophils.

### ELISA, quantitative reverse transcriptase PCR, fungal burden measurements, and flow cytometry.

Commercial ELISAs were used to measure the concentrations of albumin (Crystal Chem), hemopexin (Abcam), and (1→3)-β-d-glucan (Associates of Cape Cod), according to the manufacturers’ instructions. Heme Assay Kit (MilliporeSigma) was used for the quantification of heme; ELISA kits were used to measure hemoglobin (Abcam) and citrullinated histone-3 (Cayman Chemical) per manufacturers’ instructions. NETs were quantified by measuring neutrophil elastase–DNA complexes using an in-house ELISA, as previously described (19), using anti-mouse neutrophil elastase antibody (clone G-2, Santa Cruz Biotechnology) as capture antibody and peroxidase-conjugated anti-DNA antibody (clone MCA-33, Roche Applied Science) as detection antibody.

For quantitative reverse transcriptase PCR, liver RNA was isolated (RNeasy Plus Mini Kit, QIAGEN), and complementary DNA was synthesized using a reverse-transcription kit (QUANTAbio). Hemopexin transcript was quantified in duplicate using SYBR Green (Bio-Rad). Expression was calculated using the ΔΔCt method normalized to peptidylprolyl isomerase A, then relative to expression of uninfected animals, using commercial primers (qMmuCED0041303 and qMmuCID0016484, Bio-Rad). Reactions were performed on an iQ5 Thermal Cycler (Bio-Rad) using the following settings: 2 minutes at 95°C for activation, then 5 seconds at 95°C and 30 seconds at 60°C for 40 cycles.

Fungal CFU were determined by homogenizing freshly harvested lung tissues in 1 mL distilled water at 50 Hz for 10 minutes (TissueLyser LT, QIAGEN), followed by serial 2-fold dilutions of the samples in water and culture, in duplicate, on Sabouraud’s dextrose agar plates containing 0.05% Triton X-100. After approximately 18 hours of incubation at 37°C, plates were photographed and CFU enumerated.

Flow cytometry was performed as described previously (54, 58), using a Cytoflex LX cytometer (BD Biosciences). Briefly, cell suspensions from freshly harvested mouse lung tissue were generated by incubating mouse tissues in RPMI-1640 containing 10% FBS, liberase (MilliporeSigma), and DNase (MilliporeSigma). After lung digestion, a single-cell suspension was collected. After live-dead staining (Zombie Aqua, BioLegend) and Fc block (anti-CD16/CD32, Invitrogen), cells were stained with the following antibodies: anti-CD45–PerCP (clone 30-F11), anti-SiglecF–BV650 (clone E50-2440), and anti-CD11b–APC (clone M1/70) from BD Biosciences; anti-Ly6G–Brilliant Violet 605 (clone 1A8) and anti-CD64–PE (clone X54-5/7.1) from BioLegend, and anti-CD11c–PE–Cyanine7 (clone N418) and anti-MHCII–eFluor 450 (clone M5/114.15.2) from Invitrogen. The absolute number of each leukocyte subset in the lung was determined as the product of the percentage of that cell type and the total number of cells in the sample, enumerated with an automated cell counter (Countess 3; Invitrogen). Data were collected and analyzed using FlowJo software v. 9.0 (Tree Star).

### Computational modeling.

We expanded a previously published computational model of invasive aspergillosis (9), as described in detail in Supplemental Data 1, Supplemental Table 1, and Supplemental Table 2. Briefly, the model uses a 3-dimensional toroidal homogeneous space with a volume of 6.4 × 10^–2^ mm^3^, representing a portion of the murine lung. This space is composed of 10 × 10 × 10 voxels, with each voxel representing a 40 × 40 × 40 μm mouse alveolus, which at baseline contains type I and type II alveolar epithelial cells and alveolar macrophages. The infection in this space is initialized with 1,920 resting *Aspergillus* conidia, which scales to 10^7^ intrapulmonary conidia per mouse, the approximate inoculum size we use experimentally. After 4 hours of simulated time, the resting conidia begin to swell, then germinate into hyphae that invade the lung, resulting in recruitment of macrophages and neutrophils. When in contact with hyphae, type I alveolar epithelial cells can be either injured or killed. Type I alveolar epithelial cells can also be killed when in contact with NETs: NETs can kill any healthy cell with a low probability (<5%) and kill type I alveolar epithelial cells previously injured by hyphae with 100% probability. Death of type I alveolar epithelial cells results in filling of the alveolus with blood and release of extracellular heme. Heme is taken up by hyphae, increasing their growth. Heme contributes to hyphal growth both by providing a source of iron, and by providing the protoporphyrin ring, which independently contributes to fungal growth (5). The type I epithelial cells inhibit fungal growth in a contact-dependent manner. Recruited neutrophils can be activated by fungal (1→3)-β-d-glucan and by heme. Activated neutrophils kill *Aspergillus* and have a half-life of 6 hours, after which they undergo NETosis or apoptosis. The resulting NETs are degraded with a half-life of 3 hours (Supplemental Table 1).

### Statistics.

Data were analyzed and graphs were generated using the Prism software (version 10.4; GraphPad), and cartoons were made in BioRender (https://www.biorender.com). Differences between 2 groups at 1 time point were analyzed with an unpaired 2-tailed Mann-Whitney (nonparametric) test. Comparisons between 3 or more groups and assessment of change over time were performed using Kruskal-Wallis 1-way ANOVA with Dunn’s multiple-comparison test. Correlations were assessed using simple linear regression. Comparisons of 2 groups over time were performed using 2-way ANOVA. Survival comparisons were performed using the log-rank test. *P* values of less than 0.05 were considered statistically significant.

### Study approval.

The animal studies reported in this manuscript were approved by the Institutional Animal Care and Use Committee at the University of Florida.

### Data availability.

Individual data points are provided in the Supporting Data Values file. The computer code for the mathematical model is available at https://github.com/NutritionalLungImmunity/jISSnet/tree/master (commit ID ecef9d5).

## Author contributions

GQ, RL, and BM were responsible for conception and design. GQ, ALS, LSV, YG, MW, NGD, and AVL collected data. GQ, HALR, ALS, RL, and BM were responsible for analysis and interpretation. GQ, HALR, RL, and BM drafted the manuscript. ALS, LSV, RL, and BM performed critical revision. All authors gave final approval of the submitted version.

## Supplementary Material

Supplemental data

Supporting data values

## Version 1 04/15/2025

In-Press Preview

## Version 2 05/22/2025

Electronic publication
