# Supplementary material for: The heme scavenger hemopexin protects against lung injury during aspergillosis by mitigating release of neutrophil extracellular traps
Source: JCI Insight. 2025 Apr 15;10(10):e189151. doi: 10.1172/jci.insight.189151 (PMC12128981; doi:10.1172/jci.insight.189151)
Supplement: Supplemental data [file jciinsight-10-189151-s007.pdf]

Supplemental data for:

The heme-scavenger, hemopexin, protects against lung injury during aspergillosis by mitigating release of neutrophil-extracellular traps

Ganlin Qu, Henrique A. L. Ribeiro, Angelica L. Solomon, Luis Sordo Vieira, Yana Goddard, Nickolas G. Diodati, Arantxa V. Lazarte, Matthew Wheeler, Reinhard Laubenbacher, Borna Mehrad

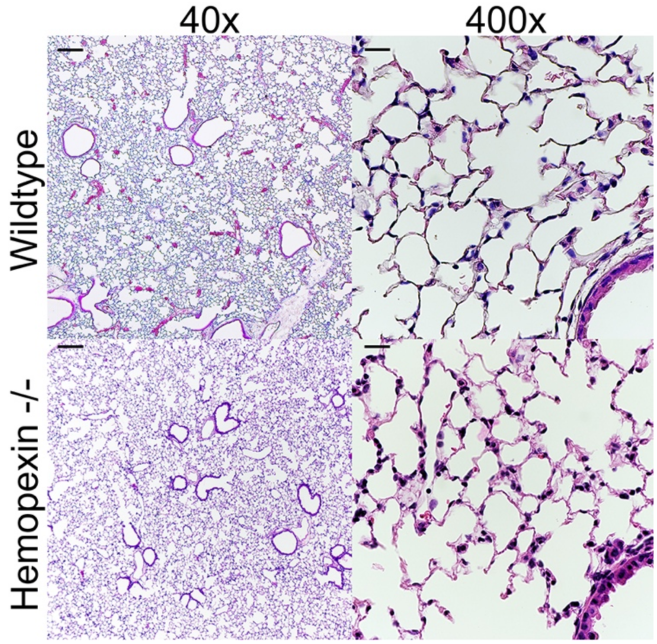

Figure S1. Lung histology (Hematoxylin and eosin stain) of uninfected wildtype and hemopexin-deficient mice. Representative images from 3 independent experiments. Original magnifications are indicated. Scale bars are 200µm and 20µm long in the 40x and 400x micrographs, respectively.

A

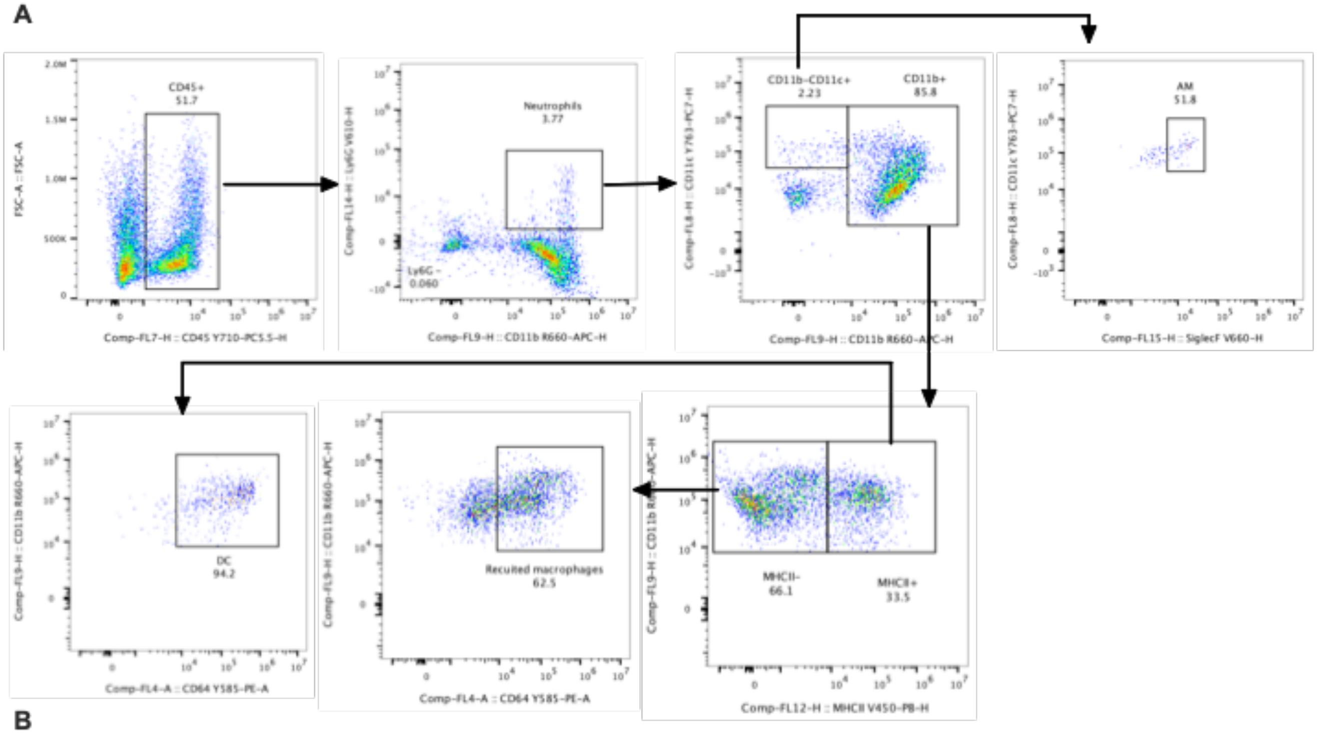

B

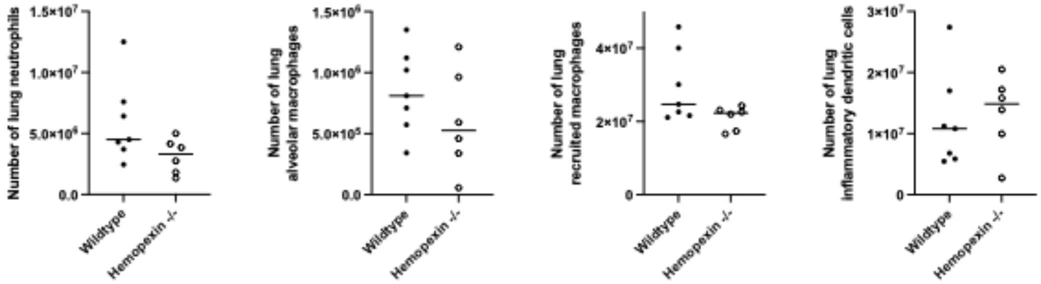

Figure S2. Lung flow cytometry on day 3 of aspergillosis. (A) Flow cytometry gating strategy of lung leukocyte subsets. Lung cells were first gated on single cells, then live cells, followed by the panels shown. (B) Absolute number of lung leukocyte subsets in wildtype and hemopexin-deficient mice on day 3 of infection. Dots represent individual animals and horizontal lines represent medians. Data shown are pooled from 2 independent experiments. No significant statistical differences by two-tailed Mann-Whitney.

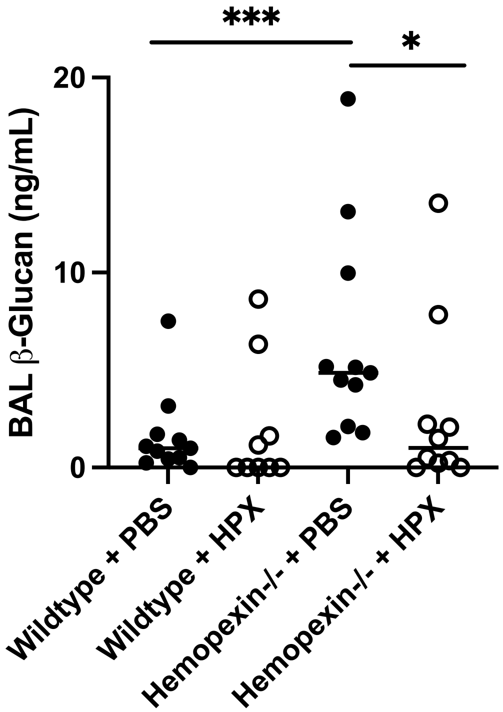

Figure S3. The effect of administration of intra-pulmonary hemopexin (HPX), as compared to PBS vehicle, on lung fungal burden (measured as BAL β-glucan concentration) on day 3 of infection. Dots represent individual animals and horizontal lines represent medians. Data shown are pooled from 2 independent experiments. * denotes *p* values of <0.05 by one-way ANOVA with Dunn's multiple comparison test.

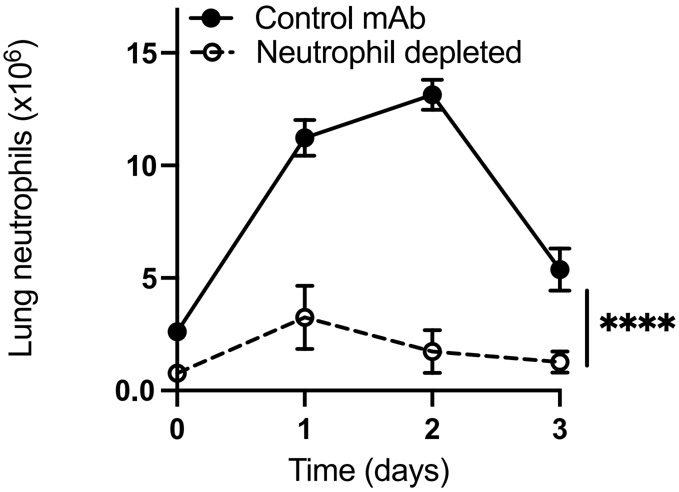

Figure S4. Number of lung neutrophils in the lung of wild type mice, after administration of neutrophil-depleting antibody (clone 1A8) or isotype control (clone 2A3) on day -1, followed by intrapulmonary challenge with *Aspergillus* conidia on day 0. Values represent mean ± SEM of n = 5 animals per group per time point, and time 0 refers to antibody-treated but uninfected animals. Data shown are pooled from 2 independent experiments. **** denotes p values of <0.0001 by two-way ANOVA.

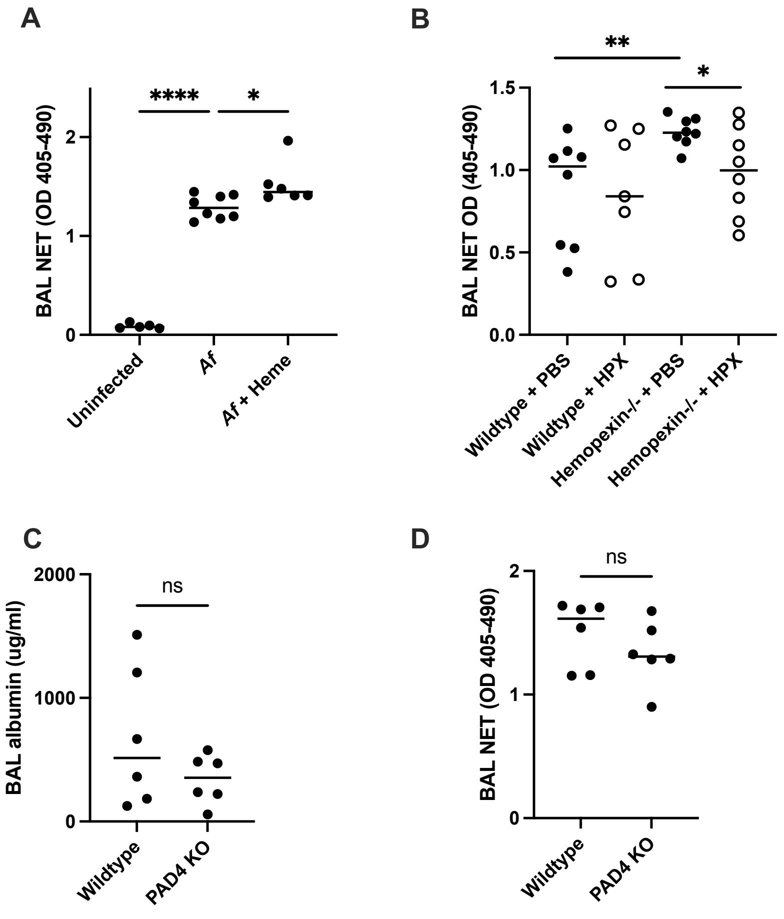

Figure S5. The effect of heme on lung NET formation in wildtype mice with neutropenic aspergillosis and the role PAD-4. BAL (A) Lung NET content of uninfected wildtype mice and *Aspergillus* infected neutropenic wildtype mice with or without administration of heme. (B) Lung NET content of neutropenic wildtype and hemopexin-deficient mice with neutropenic aspergillosis, with or without treatment with intrapulmonary hemopexin. (C-D) Extent of lung injury, as measured as BAL fluid albumin concentration and level of BAL NETs in neutropenic wildtype and PAD-4 deficient mice with aspergillosis. Dots represent individual animals and horizontal lines represent medians. Each panel represents pooled data from 2 independent experiments. *, **, and **** denote *p* values of <0.05, <0.01, and <0.0001 respectively. Statistical tests: A-B, one-way ANOVA; C-D, two-tailed Mann-Whitney.

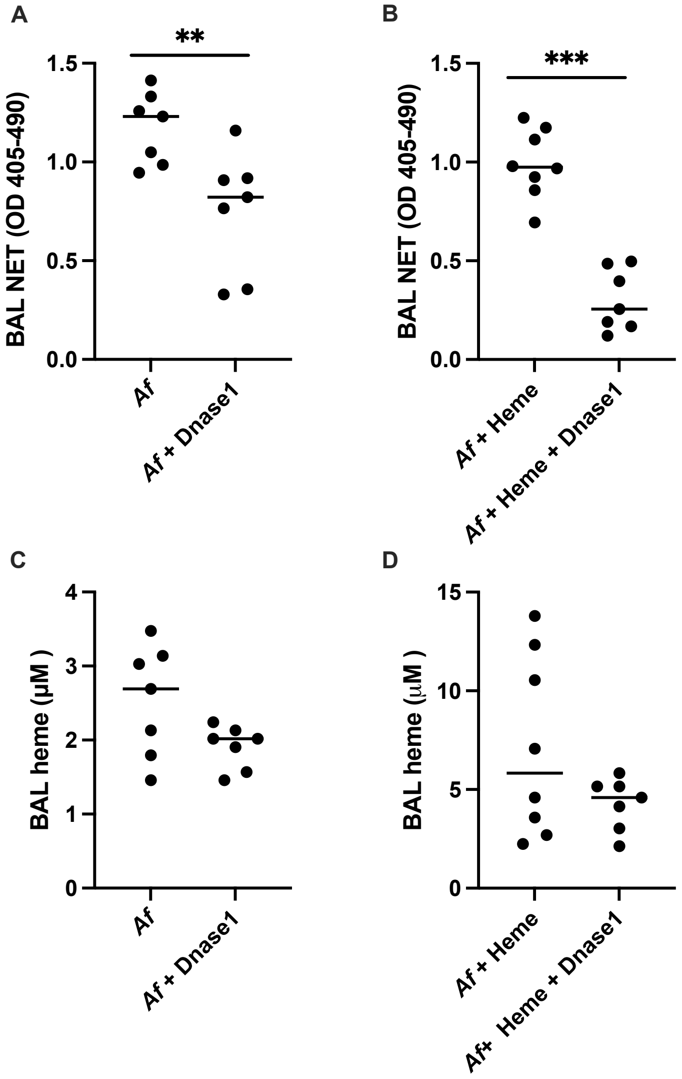

Figure S6. Effect of DNAse treatment on lung NET formation and extravascular heme in wildtype mice with neutropenic aspergillosis. (A, C) DNAse-1 was administered on day 2 and BAL NETs measured on day 3 of infection. (B, D) Dnase1 was administered on day 1 and measurements were taken on day 2 of infection. Dots represent individual animals and horizontal lines represent medians. Each panel show pooled data from 2 independent experiments. ** and *** denote *p* values of <0.01 and <0.001 respectively by two-tailed Mann-Whitney.

Computational model

Description of the base model

The base model is described in detail in [1]. We provide a general description of this model in this section for completeness: The model contains five cell types: *Aspergillus* (as resting conidia, swollen conidia, or hyphae), neutrophils, macrophages, and types I and II pneumocytes. Macrophages and neutrophils are motile cells and move randomly or biased towards a chemokine gradient, and die with a half-life (Table S1). Contact with hyphae and swollen conidia activates leukocytes and type II pneumocytes, leading them to secrete cytokines (TNF, IL10, CCL4, CXCL2). Leukocytes can phagocytose swollen conidia and kill hyphae. Type I pneumocytes inhibit hyphae elongation by a contact-mediated mechanism. Contact with the fungus and TNF each activate macrophages classically, to an M1 phenotype, whereas IL10, TGF, and apoptotic bodies activate macrophages to an M2 phenotype incapable of killing. Activated neutrophils also degranulate, releasing lactoferrin, a molecule that competes with fungal siderophore, TAFC, for iron.

Aspergillus starts as resting conidia. After four hours, it becomes swollen becomes visible for leukocytes and type II pneumocytes, and begins secreting siderophores. Both iron and heme serve as nutrients for fungal growth: with enough iron, *Aspergillus* conidia germinate, and hyphae will elongate and branch. Equation 1 computes the number of iterations for the next 40 um segment of fungi to grow, given the internal concentration of heme and iron. We use the reciprocal of Michaelean kinetics as a phenomenological equation to integrate heme and iron as nutrients that contributing to fungal growth.

$$t = T \cdot \frac{K_I \cdot I + K_H \cdot H + I \cdot H}{H \cdot I} \cdot EPI_{INH}$$

Equation 1: where "t" is the number of iterations until the next 40 um hyphal fragment grows. "T" is the inverse of the growth rate (r). K_I is the iron K_M, and K_H is the heme K_M. I and H are internal iron and heme concentrations. EPI_{INH} is the growth inhibition by alveolar epithelial cells.

Cytokines and TAFC released into the alveolus diffuse according to a partial differential equation [1], undergo decay (with a half-life), and diffuse into plasma.

Changes from the published base model

Although cells interact with their environment continuously, the experimental data that the model is based on is not continuous. In order for the model to more closely match the available experimental data, we revised the model so that each cell interacts with its environment every 30 minutes instead of every iteration. In every iteration, we select a subset of cells of a type (such as a subset of the macrophage) to interact with other cells and molecules, with any given cell is selected only every 15 iterations (30 minutes). Likewise, the Boolean networks are updated every thirty minutes.

We rewrote the state model from epithelial cells and neutrophils as Boolean networks. Neutrophils are activated by heme or *Aspergillus*. Activated neutrophils release lactoferrin and then change to either apoptotic or NETotic states, whereas non-activated neutrophils only die by apoptosis. Neutrophil apoptosis or NETosis occur with a half-life of 6h. NETs and hyphae kill type I pneumocytes, result in release of extracellular heme

into the alveolus. In addition to neutrophils, heme also activates M1 macrophages (described below). Once a type I epithelial cell dies, it results in the appearance of extracellular heme in the alveolus, simulating hemorrhage. The heme quantity is finite and is not replenished and does not diffuse, simulating clotting after hemorrhage. Once the type I pneumocytes die, their inhibition over hyphae elongation is lifted.

We refined the macrophage state model to a Boolean network model based on [104]. We ran the network until it reached equilibrium, and then assessed the macrophage phenotypes. Macrophages with activation of NFKB, STAT1, or STAT5 are classified as M1; STAT6 as M2A; ERK as M2B; and STAT3 as M2C.

Since the quantity of cytokines secreted by neutrophils was minimal in the previous model, we eliminated neutrophil cytokine secretion in this version. We also removed the IL6-hepcidin axis from the model, because this axis had little effect on the model outcome, and experimental evidence from our group showed that hepcidin did not affect infection evolution [69].

Table S1: Parameters of the revised mathematical model.

ID	Parameter	Description	*Value*	Reference
1	CCL4_QTTY	CCL4 secretion rate by macrophages and type II pneumocytes	$1.79 \times 10^{-20}\ \mathrm{mol} \cdot \mathrm{cell}^{-1} \cdot \mathrm{h}^{-1}$	[2,3,4,5,6,7,8,9, 10,11,12,13,14, 15,16,17,18,19, 20,21,22,23,24, 25,26,27,28,29, 30,31]
2	CXCL2_QTTY	CXCL2 secretion rate by macrophages and type II pneumocytes	$1.11 \times 10^{-19}\ \mathrm{mol} \cdot \mathrm{cell}^{-1} \cdot \mathrm{h}^{-1}$	
3	TNF_QTTY	TNF secretion rate by macrophage and type II pneumocytes	$3.22 \times 10^{-20}\ \mathrm{mol} \cdot \mathrm{cell}^{-1} \cdot \mathrm{h}^{-1}$	
4	IL10_QTTY	IL10 secretion rate by macrophages	$6.97 \times 10^{-22}\ \mathrm{mol} \cdot \mathrm{cell}^{-1} \cdot \mathrm{h}^{-1}$	
5	TGF_QTTY	TGF secretion rate by macrophages	$1.01 \times 10^{-21}\ \mathrm{mol} \cdot \mathrm{cell}^{-1} \cdot \mathrm{h}^{-1}$	
6	Lac_QTTY	Amount of lactoferrin	$5.36 \times 10^{-18}\ \mathrm{mol} \cdot \mathrm{cell}^{-1}$	[32]
7	Kd_CCL4	Kd of the CCL4 receptor	$180\ \mathrm{pM}$	[33]
8	Kd_CXCL2	Kd of the CXCL2 receptor	$91.667\ \mathrm{pM}$	[34,35]
9	Kd_IL10	Kd of the IL10 receptor	$140\ \mathrm{pM}$	[36,37,38,39]
10	Kd_TNF	Kd of the TNF receptor	$326\ \mathrm{pM}$	[40,41,42,43,44 ,45,46,47]
11	Kd_TGF	Kd of the TGF receptor	$26.5\ \mathrm{pM}$	[48,49,50]
12	D	Diffusion rate	$850\ \mu\mathrm{m}^2/\mathrm{min}$	[51,52]
13	λ	Cytokine half-life	$1\mathrm{h}$	[53,54,55,56,57 , 58,59]
14	TF_CONC	Transferrin concentration	$32.25\ \mu\mathrm{M}$	[60,61,62]
15	APO_TF_REL_CON	Apo-transferrin relative concentration	40%	[62, 63]
16	TFFE_REL_CON	Mono-ferric transferrin relative concentration	16.57%	
17	TFFE2_REL_CON	Di-ferric transferrin relative concentration	43.43%	
18	MA_IRON_EXP	Macrophage iron export rate	$1367.30\ \mathrm{M}^{-1} \cdot \mathrm{h}^{-1}$	[64]
19	MA_IRON_IMP	Macrophage iron import rate	$5.33 \times 10^{-12}\ \mathrm{L} \cdot \mathrm{cell}^{-1} \cdot \mathrm{h}^{-1}$	[64]
20	MA_INT_IRON	Macrophage initial internal iron concentration	$1.0086 \times 10^{-14}\ \mathrm{mol}$	[62]
21	TAFC_QTTY	TAFC secretion rate	$1.0 \times 10^{-15}\ \mathrm{mol} \cdot \mathrm{cell}^{-1} \cdot \mathrm{h}^{-1}$	[65]
22	TAFCBI_UPTAKE	uptake rate of TAFC bound to iron	$1.0 \times 10^{-12}\ \mathrm{L} \cdot \mathrm{cell}^{-1} \cdot \mathrm{h}^{-1}$	[66,67]
23	K_I	Michaelian constant for the iron substrate in Equation 1	$47.43\ \mu\mathrm{M}$	[68]
24	K_H	Michaelian constant for the heme substrate in Equation 1	$979.01\ \mathrm{nM}$	[69]
25	Kcat_Km_TAFC	Kcat/Km TAFC-Tf iron chelation reaction	$397.77\ \mathrm{M}^{-1} \cdot \mathrm{s}^{-1}$	[63,65]
26	Kcat_Km_LAC	Kcat/Km lactoferrin-Tf iron chelation reaction	$399.20\ \mathrm{M}^{-1} \cdot \mathrm{s}^{-1}$	[32]
27	r	Hyphae elongation rate	$80\ \mu\mathrm{m/h}$	[70]
28	PR_BR	Hyphae branch probability	33.3%	[70]
29	PR_SW	Conidia swelling probability	0.39%	[71,72]
30	T_SWELL	Time until conidia start swelling	$4\mathrm{h}$	[73]
31	MV_RT	Leukocyte movement rate	$1.44\ \mu\mathrm{m/min}$	[74, 75]
32	N_H_KILL	Neutrophil-hyphae killing probability	22.71%	[76,77,78,79]

33	MA_H_KILL	Macrophage-hyphae killing probability	9.85%	[80,81]
34	N_PHAG	Probability of neutrophil to phagocytose swollen conidia	14.72%	[79]
35	MA_PHAG	Probability of macrophage to phagocytose swollen conidia	90.55%	[82,83]
36	MA_MAX_CONIDIA	Maximum number of phagocytosed conidia inside a macrophage	18	[83]
37	N_MAX_CONIDIA	Maximum number of phagocytosed conidia inside a neutrophil	3	[83,84]
38	E_INT	Epithelial cell-Aspergillus (swollen conidia or hyphae) interaction probability	4.49%	[85]
39	PHAG_KILL	Leukocyte probability to kill ingested conidia	1.28%	[86]
40	MA_HALF_LIFE	Macrophage half-life	24h	[87]
41	N_HALF_LIFE	Neutrophil half-life	6h	[88]
42	PR_NET	Probability that an activated neutrophil will undergo NETosis instead of apoptosis	30%	[89]
43	H_VOL	Hyphae volume	1.06×10^{-12} L	[90,91,92, 93]
44	SEPATE_L	Length of hyphal segments between septa	40 μm	[92,93]
45	CONIDIA_VOL	Swollen conidia volume	4.84×10^{-14} L	[91]
46	MA_VOL	Macrophage volume	4.85×10^{-12} L	[94]
47	TURNOVER_RT	Molecule exchange rate between lung and whole-body serum	0.1823h^{-1}	[95]
48	MAX_N	Maximum number of neutrophils	522	[96, 97, 98, current manuscript]
49	MIN_N	Minimum number of neutrophils	15	
50	MAX_MA	Maximum number of macrophages	627	
51	MIN_MA	Minimum number of macrophages	15	
52	REC_RT	Leukocyte recruitment rate	9.77×10^{14}	[97]
53	ATII_NUM	Number of type II pneumocytes	640	[99, 100]
54	AVG_ATI_NUM	Average number of type I pneumocytes	320	
55	AF_INIT_IRON	A. fumigatus initial iron content	3.83×10^{-18} mol	[91,68]
56	AF_INIT_HEME	A. fumigatus initial heme	1.03×10^{-18} mol	[69]
57	HEME_UP	Heme uptake rate	$7.81 \times 10^{-4}\ \text{L} \cdot \text{cell}^{-1} \cdot \text{h}^{-1}$	[69, Current manuscript]
58	HEME_QTTY	Amount of heme that enters the alveoli upon hemorrhage	2.07×10^{-15} mol	Current manuscript
59	PR_NET_KILL	Probability that NET will kill a type I epithelial cell	1.3%	[101, Current manuscript]
60	PR_HYPHAE_KILL	Probability that hyphae will kill a type I epithelial cell	2.51%	[102]
61	EPI_INHIB	Type I epithelial cells rate of inhibition of hyphae elongation	50%	[71]
62	NET_HALF_LIFE	NET half-life	3h	[103]

Notes: Parameters 1-5 were obtained as described [1]. Probabilities of phagocytosis, killing, and interaction refer to the likelihood of an event succeeding in one iteration if the appropriate conditions apply. The maximum number of cells and the average number of

epithelial cells is computed over the simulated space. Kcat, Enzymatic turnover number; Kd, dissociation constant; Km, Michaelis constant; TAFC, triacetylfusarinine C (*Aspergillus* siderophore); Tf, Transferrin.

Table S2: Interaction rules in the mathematical model.

ID	Interaction	Description	Type	Outcome	Reference
1	Macrophage -Neutrophil	Macrophage phagocytose apoptotic neutrophil	Probabilistic: Constant Probability	Phosphatidyl serine receptor activated	[9]
2	Macrophage -Aspergillus	Macrophage phagocytose swollen conidia	Probabilistic: Constant Probability	Dectin-2 receptor activated; conidia are internalized and subsequently killed	[4,105,106]
3	Macrophage -Aspergillus	Macrophage kills hyphae	Probabilistic: Constant Probability	Dectin-2 receptor activated; hyphae are killed	[4,80,81]
4	Macrophage -IL10	Macrophage with phenotype M1, M2A, M2B, and M2C secrete IL10	Deterministic: fixed amount	The local concentration of IL10 increases	[6,10,14,21,104, 107]
5	Macrophage -TGF	Macrophage with phenotype M2C secrete TGF	Deterministic: fixed amount	The local concentration of TGF increases	[6,10,14,21,107]
6	Macrophage -TNF	Macrophage with phenotype M1 and M2B secrete TNF	Deterministic: fixed amount	The local concentration of TNF increases	[2,3,5,6,10, 14,21,104,107,1 08]
7	Macrophage -CCL4	Macrophage with phenotype M1 secretes CCL4	Deterministic: fixed amount	The local concentration of CCL4 increases	[2,104,107]
8	Macrophage -CXCL2	Macrophage with phenotype M1 secretes CXCL2	Deterministic: fixed amount	The local concentration of CXCL2 increases	[5,104,108]
9	Macrophage -IL10	Macrophage primed by IL10	Probabilistic: constant probability	IL10 Receptor activated	[104,109]
10	Macrophage -TGF	Macrophage primed by TGF	Probabilistic: constant probability	TGF Receptor activated	[8,9,104]
11	Macrophage -TNF	Macrophage primed by TNF	Probabilistic: constant probability	TNF Receptor activated	[27,104]
12	Macrophage -Transferrin	Macrophage import/export iron from/to transferrin	Deterministic: import and export rate are proportional to the external levels of transferrin bound to iron and internal iron levels	Internal and external levels of iron and transferrin-bound iron change.	[64,110,111,112]
13	Neutrophil-Lactoferrin	Active Neutrophils release lactoferrin	Deterministic: fixed amount	The local concentration of lactoferrin increases	[77,113]
14	Neutrophil-Aspergillus	Neutrophils phagocytose swollen conidia	Probabilistic: constant probability	Neutrophils become active; swollen conidia are internalized and subsequently killed	[77]
15	Neutrophil-Aspergillus	Neutrophils kill hyphae	Probabilistic: constant probability	Neutrophils become active; hyphae are killed	[77,105]
16	Type II Pneumocyte -Aspergillus	Type II Pneumocyte interacts with swollen conidia or hyphae	Probabilistic: initial interaction has a fixed probability. Once the	Type II Pneumocyte becomes cytokine-secreting	[114,115]

			interaction is established it is stable		
17	Type II Pneumocyte -TNF	cytokine secreting Type II Pneumocyte secrete TNF	Deterministic: fixed amount	The local concentration of TNF increases	[31]
18	Type II Pneumocyte -CCL4	Chemokine secreting Type II Pneumocyte secrete CCL4	Deterministic: fixed amount	The local concentration of CCL4 increases	[116]
19	Type II Pneumocyte -CXCL2	Chemokine secreting Type II Pneumocyte secrete CXCL2	Deterministic: fixed amount	The local concentration of CXCL2 increases	[116]
20	Type II Pneumocyte -TNF	Type II Pneumocyte is primed by TNF	Probabilistic: constant probability	Type II Pneumocyte becomes cytokine/chemokine secreting	[31]
21	Aspergillus-TAFC	Aspergillus (hyphae and swollen conidia) with TAFC node "ON" secretes TAFC	Deterministic: fixed amount	The local concentration of TAFC increases	[68,117,118]
22	Aspergillus-TAFC	Aspergillus with nodes MirB and EstB "ON" import TAFC bound to iron *	Deterministic: proportional to the concentration of TAFC bound to iron	The local concentration of TAFC-bound iron decreases. Internal iron pool increases	[66,117]
23	TAFC-transferrin	TAFC sequester iron from transferrin bound to iron	Deterministic: Michaelian kinetics	The local concentration of TAFC and Tf-iron decreases Local levels of TAFC-iron and free-transferrin increases.	[65,118]
24	Lactoferrin-transferrin	Lactoferrin sequester iron from transferrin bound to iron	Deterministic: Michaelian kinetics	The local concentration of lactoferrin and Tf-iron decreases. Local levels of lactoferrin bound to iron and free-transferrin increases.	[32,119]
25	Macrophage -iron	Necrotic macrophage releases its iron content	Deterministic: the whole iron content of the cell is released	The local concentration of iron increases	NA**
26	Iron-Aspergillus	Dead hyphae release its iron content	Deterministic: the whole iron content of the cell is released	The local concentration of iron increases	NA**
27	Iron-Iron-transport-molecule	Lactoferrin, Transferrin, or TAFC chelates the whole iron in content of the voxel	Deterministic: the molecules race to chelate the iron in the voxel. The first to be selected chelates the whole iron content or the maximum of its capacity	The local iron concentration decreases to zero. Iron bound to carriers (TAFC-iron, Lactoferrin-iron, or Tf-iron) increase.	NA
28	Type I Pneumocytes-Aspergillus	Hyphae kill type I pneumocytes. Pneumocytes that	Probabilistic: constant probability to kill	Type I cell dies or is injured	[102]

		are not killed are injured.			
29	Type I Pneumocytes-Aspergillus	Live Type I pneumocyte decrease hyphae elongation rate	Deterministic: decrease the elongation rate by a fix percentage	Elongation rate decreases	[71]
30	Type I pneumocyte-Heme	Dead type I pneumocytes releases extracellular heme in the alveolus	Deterministic	Heme become available to interact with neutrophils and Aspergillus.	This work
31	Type I-Neutrophil	NET kill type I pneumocyte	Probabilistic: constant probability (default scenario).<br>Deterministic: NET kill injured cells (alternative scenario)	Type I cell become dead	[101]
32	Heme-Aspergillus	Aspergillus uptake Heme	Deterministic: proportional to the Heme concentration	The local heme concentration decrease. Internal Iron and Heme concentration increases	[69]
33	Heme-Neutrophil	Neutrophils are primed by Heme	Probabilistic: constant probability	Neutrophils become active	[120]

* MirB and EstB respectively mediate the uptake and hydrolysis of TAFC-iron complex by *Aspergillus.*
** For simplicity, we assume that upon death, host and fungal cells release their iron in free form.
NET, neutrophil extracellular trap; TAFC, triacetylfusarinine C (*Aspergillus* siderophore); Tf, Transferrin

References

[1] Ribeiro HA, Vieira LS, Scindia Y, Adhikari B, Wheeler M, Knapp A, Schroeder W, Mehrad B, Laubenbacher R. Multi-scale mechanistic modelling of the host defence in invasive aspergillosis reveals leucocyte activation and iron acquisition as drivers of infection outcome. J R Soc Interface. 2022 Apr;19(189):20210806. doi: 10.1098/rsif.2021.0806.

[2] Werner JL, Metz AE, Horn D, Schoeb TR, Hewitt MM, Schwiebert LM, et al. Requisite Role for the Dectin-1 b- Glucan Receptor in Pulmonary Defense against Aspergillus fumigatus. The Journal of Immunology. 2009;182(8):4938–4946. doi:10.4049/jimmunol.0804250.

[3] Taylor P, Tsoni S, Willment J, et al . Dectin-1 is required for β-glucan recognition and control of fungal infection. Nat Immunol. 2007;8:31–38. doi:10.1038/ni1408.

[4] Gersuk GM, Underhill DM, Zhu L, Marr KA. Dectin-1 and TLRs Permit Macrophages to Distinguish between Different Aspergillus fumigatus Cellular States. The Journal of Immunology. 2006;176(6):3717–3724. doi:10.4049/jimmunol.176.6.3717.

[5] Hohl T, Van Epps H, Rivera A, Morgan L, Chen P, et al . Aspergillus fumigatus Triggers Inflammatory Responses by Stage-Specific b-Glucan Display. PLOS Pathogens. 2005;1(3):e30. doi:10.1371/journal.ppat.0010030.

[6] Chai L, Netea M, Sugui J, Vonk A, van de Sande W, Warris A, et al. Aspergillus fumigatus conidial melanin modulates host cytokine response. Immunobiology. 2010;215(11):915–920. doi:10.1016/j.imbio.2009.10.002.

[7] Taylor P, Roy S, Leal S, et al. Activation of neutrophils by autocrine IL-17A–IL-17RC interactions during fungal infection is regulated by IL-6, IL-23, RORγt and dectin-2. Nat Immunol. 2014;15:3007–3017. doi:10.1038/ni.2797.

[8] Celio GFdL, Xiao YQ, Shyra JG, Donna LB, William PS, Peter MH. Apoptotic Cells, through Transforming Growth Factor-β, Coordinately Induce Anti-inflammatory and Suppress Pro-inflammatory Eicosanoid and NO Synthesis in Murine Macrophages. J Biol Chem. 2006;281:38376–. doi:10.1074/jbc.M605146200.

[9] Fadok VA, Bratton DL, Konowal A, Freed PW, Westcott JY, Henson PM. Macrophages that have ingested apoptotic cells in vitro inhibit proinflammatory cytokine production through autocrine/paracrine mechanisms involving TGF-beta, PGE2, and PAF. The Journal of Clinical Investigation. 1998;101(2). doi:10.1172/JCI1112.

[10] Steele C, Rapaka R, Metz A, Pop S, Williams D, et al. The Beta-Glucan Receptor Dectin-1 Recognizes Specific Morphologies of Aspergillus fumigatus. PLOS Pathogens. 2005;1(4):e42. doi:10.1371/journal.ppat.0010042.

[11] Adachi Y, Okazaki M, Ohno N, Yadomae T. Enhancement of cytokine production by macrophages stimulated with (1 − − > 3)-beta-D-glucan, grifolan (GRN), isolated from Grifola frondosa. Biol Pharm Bull. 1994;17(12):1554–1560. doi:10.1248/bpb.17.1554.

[12] Okazaki M, Adachi Y, Ohno N, Yadomae T. Structure-activity relationship of (1–¿3)-beta-D-glucans in the induction of cytokine production from macrophages, in vitro. Biol Pharm Bull 1995 Oct;18. 10;18(10):1320–7. doi:10.1248/bpb.18.1320.

[13] Brummer E, Kamberi M, Stevens DA. Regulation by Granulocyte-Macrophage Colony-Stimulating Factor and/or Steroids Given In Vivo of Proinflammatory Cytokine and Chemokine Production by Bronchoalveolar Macrophages in Response to Aspergillus Conidia. The Journal of Infectious Diseases,. 2003;187(4):705–709. doi:10.1086/368383.

[14] Mihai GN, Warris A, Jos WMVdM, Matthew JF, Trees JGVJ, Liesbeth EHJ, et al. Aspergillus fumigatus Evades Im- mune Recognition during Germination through Loss of Toll-Like Receptor-4-Mediated Signal Transduction. The Journal of Infectious Diseases,. 2003;188(2):320–326. doi:10.1086/376456.

[15] Marika K, Elmer B, Davidm AS. Regulation of Bronchoalveolar Macrophage Proinflammatory Cytokine Production By Dexamethasone and Granulocyte-Macrophage Colony-Stimulating Factor After Stimulation By Aspergillus Conidia Or Lipopolysaccharide. Cytokine,. 2002;19(1):14–20. doi:10.1006/cyto.2002.1049.

[16] Warris A, Netea M, Verweij P, Gaustad P, Kullberg B, Weemaes C, et al. Cytokine responses and regulation of interferon- gamma release by human mononuclear cells to Aspergillus fumigatus and other filamentous fungi. Med Mycol. 2005;43(7):613– 21. doi:10.1080/13693780500088333.

[17] Fadok VA, Bratton DL, Guthrie L, Henson PM. Differential Effects of Apoptotic Versus Lysed Cells on Macrophage Pro- duction of Cytokines: Role of Proteases. J Immunol. 2001;166(11):6847–6854. doi:10.4049/jimmunol.166.11.6847.

[18] Fujishima S, Hoffman AR, Vu KJ T Kim, Zheng H, Daniel D, Kim W Y, et al. Regulation of neutrophil interleukin 8 gene expression and protein secretion by LPS, TNF-α, and IL-1β. J Cell Physiol,. 1993;154:478–485. doi:10.1002/jcp.1041540305.

[19] Xing L, Remick D. Relative cytokine and cytokine inhibitor production by mononuclear cells and neutrophils. Shock. 2003;20(1):10–6. doi:10.1097/01.shk.0000065704.84144.a4.

[20] Altstaedt J, Kirchner H, Rink L. Cytokine production of neutrophils is limited to interleukin-8. Immunology,. 1996;89:563– 568. doi:10.1046/j.1365-2567.1996.d01-784.x.

[21] Bondeson J, Browne K, Brennan F, Foxwell B, Feldmann M. Selective regulation of cytokine induction by adenoviral gene transfer of IkappaBalpha into human macrophages: lipopolysaccharide-induced, but not zymosan-induced, proinflammatory cytokines are inhibited, but IL-10 is nuclear factor-kappaB independent. J Immunol. 1999;162(5):2939–45.

[22] Abe Y, Hashimoto S, Horie T. Curcumin inhibition of inflammatory cytokine production by human peripheral blood monocytes and alveolar macrophages. Pharmacol Res. 1999;39(1):41–7. doi:10.1006/phrs.1998.0404.

[23] Loeffler J, Haddad Z, Bonin M, Romeike N, Mezger M, Schumacher U, et al. Interaction analyses of human monocytes co-cultured with different forms of Aspergillus fumigatus. Journal of Medical Microbiology. 2009;58(1):49–58. doi:10.1099/jmm.0.003293-0.

[24] Simitsopoulou M, Roilides E, Likartsis C, Ioannidis J, Orfanou A, Paliogianni F, et al. Expression of Immunomodulatory Genes in Human Monocytes Induced by

Voriconazole in the Presence of Aspergillus fumigatus. Antimicrobial Agents and Chemotherapy. 2007;51(3):1048–1054. doi:10.1128/AAC.01095-06.

[25] Lord PC, Wilmoth LM, Mizel SB, McCall CE. Expression of interleukin-1 alpha and beta genes by human blood polymorphonuclear leukocytes. The Journal of Clinical Investigation. 1991;87(4):1312–1321. doi:10.1172/JCI115134.

[26] Cassatella M. The production of cytokines by polymorphonuclear neutrophils. Immunol Today. 1995;16(1):21–6. doi:10.1016/0167-5699(95)80066-2.

[27] Ciesielski C, Andreakos E, Foxwell B, Feldmann M. TNFa-induced macrophage chemokine secretion is more dependent on NF-kB expression than lipopolysaccharides-induced macrophage chemokine secretion. European Journal of Immunology. 2002;32(7):2037–2045. doi:10.1002/1521-4141(200207)32:7¡2037::AID-IMMU2037¿3.0.CO;2-I.

[28] Palmberg L, Larsson B, Malmberg P, Larsson K. Induction of IL-8 production in human alveolar macrophages and human bronchial epithelial cells in vitro by swine dust. Thorax. 1998;53(4):260–4. doi:10.1136/thx.53.4.260.

[29] Jablonski H, Rekasi H, Jager M. The influence of calcitonin gene-related peptide on markers of bone metabolism in MG-63 osteoblast-like cells co-cultured with THP-1 macrophage-like cells under virtually osteolytic conditions. BMC Musculoskelet Disord. 2016;17(199.). doi:10.1186/s12891-016-1044-5.

[30] Katsuo K, Bo-Ram O. Optofluidic cellular immunofunctional analysis by localized surface plasmon resonance. Proc SPIE 9166, Biosensing and Nanomedicine VII, 91660R. 2014;doi:10.1117/12.2062244.

[31] Thorley AJ, Ford PA, Giembycz MA, Goldstraw P, Young A, Tetley TD. Differential Regulation of Cytokine Release and Leukocyte Migration by Lipopolysaccharide-Stimulated Primary Human Lung Alveolar Type II Epithelial Cells and Macrophages. The Journal of Immunology. 2007;178(1):463–473. doi:10.4049/jimmunol.178.1.463.

[32] Jacques LvS, Pierre LM, Joseph FH. The Involvement Of Lactoferrin In The Hyposideremia Of Acute Inflammation. J Exp Med. 1974;140(4):1068–1084. doi:10.1084/jem.140.4.1068.

[33] Samson M, LaRosa G, Libert F, Paindavoine P, Detheux M, Vassart G, et al. The second extracellular loop of CCR5 is the major determinant of ligand specificity. J Biol Chem. 1997;272(40):24934–24941. doi:10.1074/jbc.272.40.24934.

[34] Sai J, Fan GH, Wang D, Richmond A. The C-terminal domain LLKIL motif of CXCR2 is required for ligand-mediated polarization of early signals during chemotaxis. Journal of Cell Science. 2004;117(23):5489–5496. doi:10.1242/jcs.01398.

[35] Al-Alwan LA, Chang Y, Mogas A, Halayko AJ, Baglole CJ, Martin JG, et al. Differential Roles of CXCL2 and CXCL3 and Their Receptors in Regulating Normal and Asthmatic Airway Smooth Muscle Cell Migration. The Journal of Immunology. 2013;191(5):2731–2741. doi:10.4049/jimmunol.1203421.

[36] Liu Y, Wei S, Ho A, de Waal MR, Moore K. Expression cloning and characterization of a human IL-10 receptor. J Immunol. 1994;152(4):1821–9.

[37] Tan J, Indelicato S, Narula S, Zavodny P, Chou C. Characterization of interleukin-10 receptors on human and mouse cells. J Biol Chem. 1993;268(28):21053–21059.

[38] Ho AS, Liu Y, Khan TA, Hsu DH, Bazan JF, Moore KW. A receptor for interleukin 10 is related to interferon receptors. Proceedings of the National Academy of Sciences. 1993;90(23):11267–11271. doi:10.1073/pnas.90.23.11267.
[39] Carson W, Lindemann M, Baiocchi R, Linett M, Tan J, Chou C, et al. The functional characterization of interleukin-10 receptor expression on human natural killer cells. Blood. 1995;85(12):3577–85.
[40] Schall T, Lewis M, Koller K, Lee A, Rice G, Wong G, et al. Molecular cloning and expression of a receptor for human tumor necrosis factor. Cell. 1990;61(2):361–70. doi:10.1016/0092-8674(90)90816-w.
[41] Aggarwal B, Eessalu T, Hass P. Characterization of receptors for human tumour necrosis factor and their regulation by γ-interferon. Nature. 1985;318:665–667. doi:10.1038/318665a0.
[42] Tsujimoto M, Yip YK, Vilcek J. Tumor necrosis factor: specific binding and internalization in sensitive and resistant cells. Proceedings of the National Academy of Sciences. 1985;82(22):7626–7630. doi:10.1073/pnas.82.22.7626.
[43] Baglioni C, McCandless S, Tavernier J, Fiers W. Binding of human tumor necrosis factor to high affinity receptors on HeLa and lymphoblastoid cells sensitive to growth inhibition. J Biol Chem. 1985;260(25):13395–7.
[44] Tsujimoto M, vilcek J. Tumor Necrosis Factor-Induced Downregulation of Its Receptors in HeLa Cells. The Journal of Biochemistry,. 1987;102(6):1571–1577. doi:10.1093/oxfordjournals.jbchem.a122206.
[45] Stauber G, Aiyer R, Aggarwal B. Human tumor necrosis factor-alpha receptor. Purification by immunoaffinity chromatography and initial characterization. J Biol Chem. 1988;263(35):19098–104.
[46] Hohmann H, Remy R, Brockhaus M, van Loon A. Two different cell types have different major receptors for human tumor necrosis factor (TNF alpha). J Biol Chem. 1989;264(25):14927–34.
[47] Ding A, Sanchez E, Srimal S, Nathan C. Macrophages rapidly internalize their tumor necrosis factor receptors in response to bacterial lipopolysaccharide. J Biol Chem. 1989;264(7):3924–9.
[48] Massague J, Like B. Cellular receptors for type beta transforming growth factor. Ligand binding and affinity labeling in human and rodent cell lines. J Biol Chem. 1985;260(5):2636–45.
[49] Kalter VG, Brody AR. Receptors for Transforming Growth Factor-β (TGF-β) on Rat Lung Fibroblasts Have Higher Affinity for TGF-β1 than for TGF-β2. American Journal of Respiratory Cell and Molecular Biology. 1991;4(5):397–407. doi:10.1165/a jrcmb/4.5.397.
[50] Wakefield L, Smith D, Masui T, Harris C, Sporn M. Distribution and modulation of the cellular receptor for transforming growth factor-beta. J Cell Biol. 1987;105(2):965–75. doi:10.1083/jcb.105.2.965.
[51] Goodhill GJ. Diffusion in Axon Guidance. European Journal of Neuroscience. 1997;9:1414–1421. doi:10.1111/j.1460- 9568.1997.tb01496.x.
[52] Goodhill G. Mathematical guidance for axons. Trends in Neurosciences. 1998;21(6):226–231. doi:10.1016/s0166- 2236(97)01203-4.

[53] Huhn RD, Radwanski E, Gallo J, Affrime MB, Sabo R, Gonyo G, et al. Pharmacodynamics of subcutaneous recombinant human interleukin-10 in healthy volunteers. Clinical Pharmacology & Therapeutics. 1997;62(2):171–180. doi:10.1016/S0009- 9236(97)90065-5.

[54] Zahn G, Greischel A. Pharmacokinetics of tumor necrosis factor alpha after intravenous administration in rats. Dose dependence and influence of tumor necrosis factor beta. Arzneimittelforschung. 1989;39(9):1180–1182.

[55] Oliver J, Bland L, Oettinger C, Arduino M, McAllister S, Aguero S, et al. Cytokine kinetics in an in vitro whole blood model following an endotoxin challenge. Lymphokine Cytokine Res. 1993;12(2):115–120.

[56] Kuribayashi T. Elimination half-lives of interleukin-6 and cytokine-induced neutrophil chemoattractant-1 synthesized in response to inflammatory stimulation in rats. Lab Anim Res. 2018;34:80–83. doi:10.5625/lar.2018.34.2.80.

[57] Castell JV, Geiger T, Gross V, Andus T, Walter E, Hirano T, et al. Plasma clearance, organ distribution and target cells of interleukin-6/hepatocyte-stimulating factor in the rat. European Journal of Biochemistry. 1988;177(2):357–361. doi:10.1111/j.1432-1033.1988.tb14383.x.

[58] Toft A,Falahati A,Steensberg A. Source and kinetics of interleukin-6 in humans during exercise demonstrated by a minimally invasive model. Eur J Appl Physiol. 2011;111:1351–1359. doi:10.1007/s00421-010-1755-5.

[59] Wakefield L, Winokur T, Hollands R, Christopherson K, Levinson A, Sporn M. Recombinant latent transforming growth factor beta 1 has a longer plasma half-life in rats than active transforming growth factor beta 1, and a different tissue distribution. The Journal of Clinical Investigation. 1990;86(6):1976–1984. doi:10.1172/JCI114932.

[60] Tabbah S,Buhimschi C,Rodewald-Millen K,Pierson C,Bhandari V,Samuels P, et al. Hepcidin, an Iron Regulatory Hormone of Innate Immunity, is Differentially Expressed in Premature Fetuses with Early-Onset Neonatal Sepsis. Am J Perinatol. 2018;35(9):865–872. doi:10.1055/s-0038-1626711.

[61] Moran-Lev H, Weisman Y, Cohen S, Deutsch V, Cipok M, Bondar E, et al. The interrelationship between hepcidin, vitamin D, and anemia in children with acute infectious disease. Pediatr Res. 2018;84(1):62–65. doi:10.1038/s41390-018-0005-0.

[62] Parmar JH, Mendes P. A computational model to understand mouse iron physiology and disease. PLOS Computational Biology. 2019;15(1):1–28. doi:10.1371/journal.pcbi.1006680.

[63] Hissen AHT, Moore MM. Site-specific rate constants for iron acquisition from transferrin by the Aspergillus fumigatus siderophores N’,N”,N”’-triacetylfusarinine C and ferricrocin. J Biol Inorg Chem 10, 211–220. 2005;10:211–220. doi:10.1007/s00775-005-0630-z.

[64] Sarkar J, Seshadri V, Tripoulas N, Ketterer M, Fox P. Role of ceruloplasmin in macrophage iron efflux during hypoxia. J Biol Chem. 2003;278(45):44018–24. doi:10.1074/jbc.M304926200.

[65] Hissen AHT, Chow JMT, Pinto LJ, Moore MM. Survival of Aspergillus fumigatus in Serum Involves Removal of Iron from Transferrin: the Role of Siderophores. INFECTION AND IMMUNITY. 2004;72(3):1402–1408.
[66] Raymond-Bouchard I, Carroll CS, Nesbitt JR, Henry KA, Pinto LJ, Moinzadeh M, et al. Structural Requirements for the Activity of the MirB Ferrisiderophore Transporter of Aspergillus fumigatus. Eukaryotic Cell. 2012;11(11). doi:10.1128/EC.00159- 12.
[67] Yap PY, Trau D. Direct Yeast Cell Count At OD600. Tip Biosystems Pte Ltd. 2019;2019.
[68] Schrettl M, Kim HS, Eisendle M, Kragl C, Nierman WC, Heinekamp T, et al. SreA-mediated iron regulation in Aspergillus fumigatus. Molecular Microbiology,. 2008;70:27–43. doi:10.1111/j.1365-2958.2008.06376.x.
[69] Michels K, Solomon AL, Scindia Y, Sordo Vieira L, Goddard Y, Whitten S, Vaulont S, Burdick MD, Atkinson C, Laubenbacher R, Mehrad B. Aspergillus Utilizes Extracellular Heme as an Iron Source During Invasive Pneumonia, Driving Infection Severity. J Infect Dis. 2022 May 16;225(10):1811-1821. doi: 10.1093/infdis/jiac079.
[70] Baltussen TJH, van Rhijn N, Coolen JPM, Dijksterhuis J, Verweij PE, Bromley MJ, Melchers WJG. The C2H2 transcription factor SltA is required for germination and hyphal development in Aspergillus fumigatus. mSphere. 2023 Aug 24;8(4):e0007623. doi: 10.1128/msphere.00076-23.
[71] Gago S, Overton NLD, Ben-Ghazzi Nt. Lung colonization by Aspergillus fumigatus is controlled by ZNF77. Nat Commun 9,. 2018;3835. doi:10.1038/s41467-018-06148-7.
[72] White LO. Germination of Aspergillus fumigatus conidia in the lungs of normal and cortisone-treated mice. Sabouraudia,. 1977;Volume 15, Issue 1,:37–41. doi:10.1080/00362177785190071. \
[73] Schrettl M, Bignell E, Kragl C, Sabiha Y, Loss O, Eisendle M, et al. Distinct roles for intra- and extracellular siderophores during Aspergillus fumigatus infection. PLoS Pathog. 2007;3(9):1195–207. doi:10.1371/journal.ppat.0030128.
[74] Khandoga A, Khandoga A, Reichel C, Bihari P, Rehberg M, et al . In Vivo Imaging and Quantitative Analysis of Leukocyte Directional Migration and Polarization in Inflamed Tissue. PLoS ONE. 2009;4(3):e4693. doi:10.1371/journal.pone.0004693.
[75] Barros-Becker F, Lam PY, Fisher R, Huttenlocher A. Live imaging reveals distinct modes of neutrophil and macrophage migration within interstitial tissues. J Cell Sci. 2017 Nov 15;130(22):3801-3808. doi: 10.1242/jcs.206128.
[76] Zarember K, Sugui J, Chang Y, Kwon-Chung K, Gallin J. Human polymorphonuclear leukocytes inhibit Aspergillus fumigatus conidial growth by lactoferrin-mediated iron depletion. J Immunol. 2007;178(10):6367–6373. doi:10.4049/jimmunol.178.10.6367.
[77] Gazendam RP, van Hamme JL, Tool AT, Hoogenboezem M, van den Berg JM, Prins JM, et al. Human Neutrophils Use Different Mechanisms To Kill Aspergillus fumigatus Conidia and Hyphae: Evidence from Phagocyte Defects. The Journal of Immunology. 2016;196(3):1272–1283. doi:10.4049/jimmunol.1501811.
[78] Gazendam R, van de Geer A, van Hamme J, Tool A, van Rees D, Aarts C, et al. Impaired killing of Candida albicans by granulocytes mobilized for transfusion

purposes: a role for granule components. Haematologica. 2016;101(5):587–96. doi:10.3324/haematol.2015.136630.
[79] Roilides E, Dimitriadou-Georgiadou A, Sein T, Kadiltsoglou I, Walsh T. Tumor necrosis factor alpha enhances antifungal activities of polymorphonuclear and mononuclear phagocytes against Aspergillus fumigatus. Infect Immun. 1998;66(12):5999– 6003. doi:10.1128/IAI.66.12.5999-6003.1998.
[80] Roilides E, Sein T, Holmes A, Chanock S, Blake C, Pizzo P, et al. Effects of macrophage colony-stimulating factor on antifungal activity of mononuclear phagocytes against Aspergillus fumigatus. J Infect Dis. 1995;172(4):1028–1034. doi:10.1093/infdis/172.4.1028.
[81] Roilides E, Holmes A, Blake C, Venzon D, Pizzo P, Walsh T. Antifungal activity of elutriated human monocytes against Aspergillus fumigatus hyphae: enhancement by granulocyte-macrophage colony-stimulating factor and interferon-gamma. J Infect Dis. 1994;170(4):894–9. doi:10.1093/infdis/170.4.894.
[82] Philippe B, Ibrahim-Granet O, Pr´evost M, Gougerot-Pocidalo M, Sanchez PM, Van der Meeren A, et al. Killing of Aspergillus fumigatus by alveolar macrophages is mediated by reactive oxidant intermediates. Infect Immun. 2003;71(6):3034–42. doi:10.1128/iai.71.6.3034-3042.2003.
[83] Gresnigt MS, Becke rKL, Leenders F, Alonso MF, Wang X, Meis JF, et al. Differential Kinetics of Aspergillus nidulans and Aspergillus fumigatus Phagocytosis. J Innate Immun. 2018;10:145–160. doi:10.1159/000484562.
[84] Niemiec M, De Samber B, Garrevoet J, Vergucht E, Vekemans B, De Rycke R, et al. Trace element landscape of resting and activated human neutrophils on the sub-micrometer level. Metallomics. 2015;7(6):996–1010. doi:10.1039/c4mt00346b.
[85] Clark HR, Powell AB, Simmons KA, Ayubi T, Kale SD. Endocytic Markers Associated with the Internalization and Processing of Aspergillus fumigatus Conidia by BEAS-2B Cells. mSphere. 2019;4(1):e00663–18. doi:10.1128/mSphere.00663-18.
[86] Wasylnka J, Hissen A, Wan A, Moore M. Intracellular and extracellular growth of Aspergillus fumigatus. Med Mycol. 2005;43:S27–S30. doi:10.1080/13693780400029247.
[87] Patel A, Zhang Y, Fullerton J, Boelen L, Rongvaux A, Maini A, et al. The fate and lifespan of human monocyte subsets in steady state and systemic inflammation. The Journal of experimental medicine. 2017;214. doi:10.1084/jem.20170355.
[88] Tak T, Tesselaar K, Pillay J, Borghans J, Koenderman L. What's your age again? Determination of human neutrophil half-lives revisited. J Leukoc Biol. 2013;94(4):595–601. doi:10.1189/jlb.1112571.
[89] Huang, M.YY., Lippuner, C., Schiff, M. et al. Neutrophil extracellular trap formation during surgical procedures: a pilot study. Sci Rep 13, 15217 (2023). https://doi.org/10.1038/s41598-023-42565-5
[90] Bakken L, Olsen R. Buoyant densities and dry-matter contents of microorganisms: conversion of a measured biovolume into biomass. Appl Environ Microbiol. 1983;45(4):1188–1195. doi:10.1128/AEM.45.4.1188-1195.1983.

[91] Sugui J, Kwon-Chung K, Juvvadi P, Latǵe J, Steinbach W. Aspergillus fumigatus and related species. Cold Spring Harb Perspect Med. 2014;5(2):a019786. doi:10.1101/cshperspect.a019786.
[92] Ding Z, Li M, Sun F, Xi P, Sun L, Zhang L, et al. Mitogen-activated protein kinases are associated with the regulation of physiological traits and virulence in Fusarium oxysporum f. sp. cubense. PLoS One. 2015;10(4):e0122634. doi:10.1371/journal.pone.0122634.
[93] Renshaw H, Vargas-Mun˜iz J, Juvvadi P, Richards A, Waitt G, Soderblom E, et al. The tail domain of the Aspergillus fumigatus class V myosin MyoE orchestrates septal localization and hyphal growth. J Cell Sci. 2018;131(3):jcs205955. doi:10.1242/jcs.205955.
[94] Fritz K, Silvia M, Anne-Marie A, Gerlach JT, Ju¨rgen B, Martina D. Cell Size of Alveolar Macrophages: An Interspecies Comparison. Environmental Health Perspectives. 1997;105(5):1261–1263. doi:10.2307/3433544.
[95] Goncalves SM, Lagrou K, Rodrigues CS, Campos CF, Bernal-Martinez L, Rodrigues F, et al. Evaluation of Bronchoalveolar Lavage Fluid Cytokines as Biomarkers for Invasive Pulmonary Aspergillosis in At-Risk Patients. Frontiers in Microbiology. 2017;8:2362. doi:10.3389/fmicb.2017.02362.
[96] Werner JL, Gessner MA, Lilly LM, Nelson MP, Metz AE, Horn D, Dunaway CW, Deshane J, Chaplin DD, Weaver CT, Brown GD, Steele C. Neutrophils produce interleukin 17A (IL-17A) in a dectin-1- and IL-23-dependent manner during invasive fungal infection. Infect Immun. 2011 Oct;79(10):3966-77. doi: 10.1128/IAI.05493-11.
[97] Bonnett CR, Cornish EJ,Harmsen AG,Burritt JB.Early Neutrophil Recruitment and Aggregation in the Murine Lung Inhibit Germination of Aspergillus fumigatus Conidia. Infection and Immunity. 2006;74(12):6528–6539. doi:10.1128/IAI.00909-06.
[98] Morgenstern DE, Gifford MA, Li LL, Doerschuk CM, Dinauer MC. Absence of respiratory burst in X-linked chronic granulomatous disease mice leads to abnormalities in both host defense and inflammatory response to Aspergillus fumigatus. J Exp Med. 1997 Jan 20;185(2):207-18. doi: 10.1084/jem.185.2.207.
[99] Dzhuraev G, Rodriguez-Castillo J, Ruiz-Camp J, Salwig I, Szibor M, Vadasz I, et al. Estimation of absolute number of alveolar epithelial type 2 cells in mouse lungs: a comparison between stereology and flow cytometry. Journal of Microscopy. 2019;275:36–50. doi:10.1111/jmi.12800.
[100] Crapo JD, Barry BE, Gehr P, Bachofen M, Weibel ER. Cell number and cell characteristics of the normal human lung. Am Rev Respir Dis. 1982 Aug;126(2):332-7. doi: 10.1164/arrd.1982.126.2.332. PMID: 7103258.
[101] Saffarzadeh M, Juenemann C, Queisser MA, Lochnit G, Barreto G, Galuska SP, Lohmeyer J, Preissner KT. Neutrophil extracellular traps directly induce epithelial and endothelial cell death: a predominant role of histones. PLoS One. 2012;7(2):e32366. doi: 10.1371/journal.pone.0032366.
[102] Wächtler B, Citiulo F, Jablonowski N, Förster S, Dalle F, Schaller M, Wilson D, Hube B. Candida albicans-epithelial interactions: dissecting the roles of active penetration, induced endocytosis and host factors on the infection process. PLoS One. 2012;7(5):e36952. doi: 10.1371/journal.pone.0036952.

[103] Hakkim A, Fürnrohr BG, Amann K, Laube B, Abed UA, Brinkmann V, Herrmann M, Voll RE, Zychlinsky A. Impairment of neutrophil extracellular trap degradation is associated with lupus nephritis. Proc Natl Acad Sci U S A. 2010 May 25;107(21):9813-8. doi: 10.1073/pnas.0909927107.
[104] Palma A, Jarrah AS, Tieri P, Cesareni G, Castiglione F. Gene Regulatory Network Modeling of Macrophage Differentiation Corroborates the Continuum Hypothesis of Polarization States. Front Physiol. 2018 Nov 27;9:1659. doi: 10.3389/fphys.2018.01659.
[105] Schaffner A, Douglas H, Braude A. Selective protection against conidia by mononuclear and against mycelia by polymorphonuclear phagocytes in resistance to Aspergillus. Observations on these two lines of defense in vivo and in vitro with human and mouse phagocytes. J Clin Invest. 1982;69(3):617–631. doi:10.1172/jci110489.
[106] Ibrahim-Granet O, Philippe B, Boleti H, Boisvieux-Ulrich E, Grenet D, Stern M, et al. Phagocytosis and Intracellular Fate of Aspergillus fumigatus Conidia in Alveolar Macrophages. Infection and Immunity. 2003;71(2):891–903. doi:10.1128/IAI.71.2.891-903.2003.
[107] Smedman C, G˚ardlund B, Nihlmark K, Gille-Johnson P, Andersson J, Paulie S. ELISpot analysis of LPS-stimulated leukocytes: Human granulocytes selectively secrete IL-8, MIP-1β and TNF-α. Journal of Immunological Methods. 2009;346(1):1–8. doi:10.1016/j.jim.2009.04.001.
[108] Meier A, Kirschning CJ, Nikolaus T, Wagner H, Heesemann J, Ebel F. Toll-like receptor (TLR) 2 and TLR4 are essential for Aspergillus-induced activation of murine macrophages. Cellular Microbiology. 2003;5(8):561–570. doi:10.1046/j.1462-5822.2003.00301.x.
[109] Raychaudhuri B, Fisher CJ, Farver CF, Malur A, Drazba J, Kavuru MS, et al. INTERLEUKIN 10 (IL-10)-MEDIATED INHIBITION OF INFLAMMATORY CYTOKINE PRODUCTION BY HUMAN ALVEOLAR MACROPHAGES. Cytokine. 2000;12(9):1348–1355. doi:10.1006/cyto.2000.0721.
[110] Soares M, Hamza I. Macrophages and Iron Metabolism. Immunity. 2016;44(3):1348–1355. doi:10.1016/j.immuni.2016.02.016.
[111] Fleming M, Andrews N. Mammalian iron transport: an unexpected link between metal homeostasis and host defense. J Lab Clin Med. 1998;132(6):464–8. doi:10.1016/s0022-2143(98)90123-8.
[112] Dautry-Varsat A, Ciechanover A, Lodish HF. pH and the recycling of transferrin during receptor-mediated endocytosis. Proceedings of the National Academy of Sciences. 1983;80(8):2258–2262. doi:10.1073/pnas.80.8.2258.
[113] Zaremberea KA. Human polymorphonuclear leukocytes inhibit Aspergillus fumigatus conidial growth by lactoferrin-mediated iron depletion. J Immunol. 2007;178:6367–73.
[114] Chen F, Zhang C, Jia X, Wang S, Wang J, Chen Y, et al. Transcriptome Profiles of Human Lung Epithelial Cells A549 Interacting with Aspergillus fumigatus by RNA-Seq. PLoS ONE. 2015;10(8):e0135720. doi:10.1371/journal.pone.0135720.
[115] Bellanger AP, Millon L, Khoufache K, Rivollet D, Bi`eche I, Laurendeau I, et al. Aspergillus fumigatus germ tube growth and not conidia ingestion induces expression of inflammatory mediator genes in the human lung epithelial cell line

A549. Journal of Medical Microbiology. 2009;58(2):174–179. doi:10.1099/jmm.0.005488-0.
[116] Sharma AK, Fernandez LG, Awad AS, Kron IL, Laubach VE. Proinflammatory response of alveolar epithelial cells is enhanced by alveolar macrophage-produced TNF-α during pulmonary ischemia-reperfusion injury. American Journal of Physiology-Lung Cellular and Molecular Physiology. 2007;293(1):L105–L113. doi:10.1152/ajplung.00470.2006.
[117] Brandon M, Howard B, Lawrence C, Laubenbacher R. Iron acquisition and oxidative stress response in Aspergillus fumigatus. BMC systems biology. 2015;9(1):19.
[118] Schrettl M, Bignell E, Kragl C, Joechl C, Rogers T, Arst J Herbert N, et al. Siderophore Biosynthesis But Not Reductive Iron Assimilation Is Essential for Aspergillus fumigatus Virulence. J Exp Med. 2004;200(9):1213–1219. doi:10.1084/jem.20041242.
[119] Mazurier J, Spik G. Comparative study of the iron-binding properties of human transferrins: I. Complete and sequential iron saturation and desaturation of the lactotransferrin. Biochimica et Biophysica Acta (BBA) - General Subjects. 1980;629(2):399–408. doi:10.1016/0304-4165(80)90112-9.
[120] Chen G, Zhang D, Fuchs TA, Manwani D, Wagner DD, Frenette PS. Heme-induced neutrophil extracellular traps contribute to the pathogenesis of sickle cell disease. Blood. 2014 Jun 12;123(24):3818-27. doi: 10.1182/blood-2013-10-529982.
